# Gastrointestinal Tract Remodeling by Dietary Polysaccharides Mechanistic Insights in Colitis—A Review

**DOI:** 10.3390/foods15132267

**Published:** 2026-06-24

**Authors:** Afifa Aziz, Muhammad Zeeshan Adil, Muqadas Altaf, Min Wang, Kit-Leong Cheong

**Affiliations:** 1 Guangdong Provincial Key Laboratory of Aquatic Product Processing and Safety, Guangdong Ocean University, Zhanjiang 524088, China; afifaaziz44@gmail.com (A.A.); muqadasaltaf304@gmail.com (M.A.); 2 Guangdong Province Engineering Laboratory for Marine Biological Products, Guangdong Ocean University, Zhanjiang 524088, China; 3 Guangdong Provincial Engineering Technology Research Center of Seafood, Guangdong Ocean University, Zhanjiang 524088, China; 4 Guangdong Provincial Engineering Technology Research Center of Prefabricated Seafood Processing and Quality Control, Guangdong Ocean University, Zhanjiang 524088, China; 5 College of Food Science and Technology, Guangdong Ocean University, Zhanjiang 524088, China; 6 School of Food Science and Engineering, South China University of Technology, Guangzhou 510641, China; mrzeeshan0300@gmail.com; 7 College of Coastal Agriculture Sciences, Guangdong Ocean University, Zhanjiang 524088, China

**Keywords:** bioactive polysaccharides, functional foods, gut microbiota, structure-function relationship, colitis, modification, immune regulation, oxidative stress

## Abstract

The increased global prevalence of inflammatory bowel disease (IBD), including ulcerative colitis (UC) and Crohn’s disease (CD), is a chronic relapsing inflammatory condition of the gastrointestinal tract that creates a substantial socioeconomic burden. Existing pharmacotherapeutic treatments primarily target inflammatory signaling cascades and have disadvantages because of the side effects of drugs, reduced long-term efficacy, and high cost, necessitating the development of safe and sustainable adjunctive therapies. This review synthesizes mechanistic advances regarding dietary polysaccharides as bioactive agents that may have the capacity to induce remodeling of inflamed gastrointestinal tract in colitis and could be an adjunctive strategy as functional food ingredients due to their various biological activities in the management of colitis. Polysaccharides alleviate colitis through several interconnected pathways. First, they correct the gut dysbiosis by enriching beneficial taxa such as *Lactobacillus*, *Bifidobacterium*, and *Akkermansia muciniphila*. Second, fermentation of polysaccharides produces short-chain fatty acids (SCFAs), particularly butyrate, which serve as the primary energy source for colonocytes. Third, they restore intestinal barrier integrity by upregulating tight junction proteins such as ZO-1, occludin, and claudin, also performing pro-inflammatory cascade inhibition and elimination of oxidative stress via Nrf2/HO-1 activation The relationship between structural properties of polysaccharides based on molecular weight, monosaccharide composition, and biological functions of chemically modified dietary polysaccharides in colitis is studied. Dietary polysaccharides are explored here not as replacements for pharmacotherapy but as potential adjunctive or functional food-based interventions that may complement existing treatments as safe, multitargeted, and cost-effective interventions in prevention or long-term management of colitis and IBD. This review presents dietary polysaccharides function not as passive dietary fibers but as bioactive, multi-targeted, structurally dependent agents capable of restoring intestinal homeostasis, suggesting them as potentially safe, adjunctive interventions.

## 1. Introduction

IBD, which includes ulcerative colitis and Crohn’s disease, is a chronic, relapsing inflammatory disease of the gastrointestinal (GI) tract that is a growing international health issue. Over the last few decades, the incidence and prevalence of IBD have increased dramatically across most of the world, including newly industrialized countries and younger population groups [1,2]. Overall, a huge socioeconomic burden has accompanied this epidemiological transition because of frequent hospitalizations, extended drug treatment, poor quality of life, and increased health-care spending [3].

Pathologically, colitis is manifested by continuous inflammation of the mucosa, epithelial damage, and immune defects. Hallmarks are dysbiosis of the gut microbiota, loss of the intestinal barrier, excess production of pro-inflammatory cytokines, oxidative stress, and abnormal activation of the innate and adaptive immune responses [2,4]. Repeated waves of tissue injury and repair induced by continued inflammation eventually result in architectural changes like crypt distortion, fibrosis, shortening of the colon and, in the most critical cases, resistance to therapy [1].

In this review, plant source-derived dietary polysaccharides are the study interest as safe, multi-target agents that regulate host–microbe interactions, strengthen intestinal barriers, and control immune and oxidative stress in colitis [2,5]. Dietary polysaccharides are complex biopolymers consisting of units of monosaccharides and are bound by various glycosidic bonds, display linear or branched structures, and show a high degree of structural heterogeneity [6]. The main characteristic of dietary polysaccharides is the lack of digestibility by digestive enzymes that permits them to reach the colon relatively intact, where they predominantly interact with the gut microbiota [7]. Polysaccharides act as prebiotics that selectively stimulate the growth of useful microbes, e.g., *Lactobacillus* and *Bifidobacterium*, and inhibit harmful taxa, thus normalizing dysbiotic situations [2,5].

Microbial modulation increases the generation of short-chain fatty acids (SCFAs), particularly butyrate, propionate, and acetate, which are majorly involved in intestinal homeostasis [2,5]. SCFAs are a significant source of energy available to the colonocytes; they reinforce epithelial tight junctions and have strong anti-inflammatory effects, inhibiting nuclear factor-kappa B (NF-κB) signaling, as well as inhibiting the production of pro-inflammatory cytokines [4]. There is experimental evidence that polysaccharides of various sources—*Polygonatum kingianum*, *Agrocybe cylindracea*, *Lyophyllum decastes*, ginseng, and Kombucha polysaccharides—effectively modulate colitis by remodeling the makeup of intestinal microbiota and metabolic products [8,9,10].

In addition to microbial modulation, dietary polysaccharides are significant in restoring intestinal barrier integrity, which is one of the pathological features of colitis [4]. The intestinal barrier comprises epithelial cells, tight junction proteins, layers of mucus, immune elements, as well as native microbiota. In colitis, intestinal hyperpermeability (what is known as leaky gut) is brought about by tight junction disruption, reduction in claudin expression, and loss of tight junction characters, defined by a decreased expression of proteins such as zonula occludens-1 (ZO-1), occludin and claudin [4,11]. Dietary polysaccharides have a strong correlation between their biological activity and structural properties, such as molecular weight, compositions with monosaccharide units, the degree of branching, and functional modifications (e.g., sulfation or acetylation) of the molecules [12,13,14]. There is an emerging body of evidence to indicate that low-molecular-weight polysaccharides and certain motifs might have an increased bioactivity and can be used to selectively regulate microbial, epithelial and immune pathways [12]. However, it is critical to note that not all polysaccharides are uniformly beneficial. High-molecular-weight, insoluble polysaccharides may exacerbate symptoms in active disease, while low-molecular-weight, soluble fractions show greater efficacy. Furthermore, effects are highly context-dependent, varying with polysaccharide structure, dosage, host microbiota composition, and disease stage [15].

Previous studies have individually examined the effects of dietary polysaccharides on gut microbiota modulation, immune regulation, or intestinal barrier protection in colitis as separate domains. This study distinguishes itself from prior work in the integrative “remodeling” framework, which positions dietary polysaccharides not merely as functional food, anti-inflammatory, or prebiotic agents, but as multi-compartment modulators rather than single-mechanism agents. We explain that dietary polysaccharides simultaneously target epithelial repair, immune–stromal coordination, goblet cell restoration, tight junction reassembly, extracellular matrix reorganization, anti-fibrotic remodeling and mucosal architecture restoration. This framework positions dietary polysaccharides as multi-compartment modulators rather than single-mechanism agents. Furthermore, we explicitly map how specific structural features of polysaccharide molecular weight, degree of branching (DB), monosaccharide composition, and chemical modifications (sulfation, acetylation) determine their efficacy across each remodeling axis, which may provide a structure-guided roadmap for future therapeutic design.

## 2. Colitis and Gastrointestinal Remodeling

Gastrointestinal remodeling is defined as the active, multi-compartment restructuration of inflamed intestinal tissue toward homeostatic architecture and normal function, encompassing gastrointestinal epithelial remodeling, mucus/goblet cell remodeling and extracellular matrix (ECM) remodeling [16]. Gastrointestinal epithelial remodeling is characterized by crypt regeneration, normalization of intestinal stem cell differentiation, and restoration of a functional enterocyte monolayer. The mucus/goblet cell remodeling involves the recovery of goblet cell density, normalization of Mucin 2 (MUC2) secretion, and reconstitution of a protective mucus bilayer. Additionally, extracellular matrix (ECM) remodeling rebalances matrix metalloproteinase (MMP) and microbiota dysbiosis on the extra-extracellular matrix and tissue structure; these activities resolve pathological degradation, limit excessive collagen deposition, and prevent fibrotic stricture formation [17,18,19].

**Figure 1:**
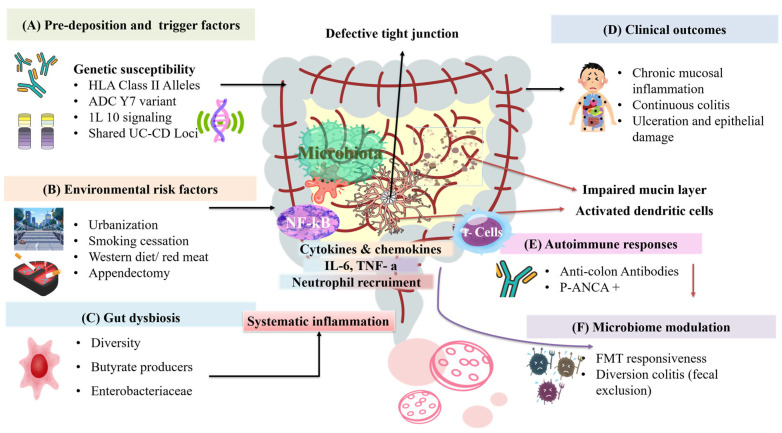
Schematic overview of the multifactorial pathogenesis of inflammatory bowel disease (IBD) and the potential point of intervention for microbiome modulation. (**A**) Pre-deposition and trigger factors initiate disease susceptibility, which interacts with (**B**) environmental risk factors (e.g., diet, antibiotics, stress) to promote gut inflammation (**C**) Gut dysbiosis, characterized by microbial imbalance, activates immune cells including T cells and promotes the release of pro-inflammatory cytokines and chemokines (e.g., IL-6, TNF-α). This cascade also involves NF-κB signaling and neutrophil recruitment, leading to (**D**) clinical outcomes such as systemic inflammation and tissue damage. Subsequently, (**E**) autoimmune responses further sustain chronic inflammation. Finally, (**F**) microbiome modulation (e.g., via dietary polysaccharides, prebiotics, or fecal microbiota transplantation) represents a therapeutic strategy aimed at restoring microbial equilibrium and breaking the inflammatory cycle.

IBDs include UC and CD, which are immune-mediated conditions of high prevalence in developed nations (>0.3%) and demonstrate a rapidly growing incidence in newly industrialized countries [20]. Global prevalence of UC is projected to reach 30 million cases by 2026. Despite the presence of several consistent clinical and pathological features that serve as strong indicators of the disease, the precise pathogenesis of UC remains incompletely defined [1,2]. UC is an a etiologically unknown disease, which is associated with the inflammation of mucosa and sub-mucosa of the colon and rectum lining, presenting diarrhea, urgency, and abdominal pain [21,22]. Its pathogenesis is associated with genetic predisposition, epithelial barrier defects, a dysregulated immune response, and environmental factors [23] as described in Figure 1.

### 2.1. Key Remodeling Mechanisms in Colitis

#### 2.1.1. Gastrointestinal Epithelial Cell Remodeling

Chronic inflammation in UC induces persistent alterations in goblet cells (GCs), resulting in abnormal secretion of mucus. Substantial evidence indicates that the reduced amount of mucus secreted by the GCs affects the mucosal state and affects UC pathophysiology due to immune responses and bacterial exposure. UC patients, even in remission, exhibit a deficient mucus layer with histological anomalies including branching of the crypts and the modification of gene expression, stimulating irreversible alterations in the intestinal lining. The systematic modifications can result in recurrent disease episodes, meaning that mucosal alterations take longer to recover after the inflammation settles down. The cause of the abnormal mucus cover in UC patients is the impact of an active inflammatory environment that diminishes the number of GCs and is also caused by long-term alterations in stimulated mucin secretion, which remain even without the presence of inflammatory cells [18]. Similarly, colitis disrupts intestinal stem cell dynamics, impairing epithelial regeneration. The additional differentiation of intestinal stem cells into GCs is controlled by cues such as fibroblast growth factor 1 (FGF1), and the weaker the FGF1 expression, the worse the disease and the loss of the intestinal integrity in the models of IBD and patients. Collectively, these findings support the fact that chronic inflammation suppresses stem and progenitor cell-meditated epithelial homeostasis, thereby contributing to persistent mucosal defects [24].

#### 2.1.2. Extracellular Matrix Remodeling

IBD is an inflammatory condition resulting in tissue degradation and fibrosis, as well as excess activity of matrix metalloproteinases, which overly breaks down components of the extracellular matrix (ECM). Intestinal remodeling is a vital component of IBD pathogenesis and disease progression. During this process, hyaluronic acid accumulates in the extracellular matrix and undergoes fragmentation, producing bioactive HA fragments that participate in wound healing and may contribute to the development of intestinal fibrosis. CM variation affects cellular pathways and triggers signaling, which affect cellular immune regulation and physiology [17]. This is an imbalance of ECM turnover that is supported by the investigations of serum biomarkers of collagen degradation products and markers of its formation. Indicatively, moderate to severe quality of the disease was shown to be correlated with high concentrations of type III and IV collagen degradation and formation indicators (C3M and C4M), also formation indicators (PRO-C3), and a combination of the two indicators has great diagnostic power (area under the ROC curve = 0.94 in UC) [25]. These statistics indicate that ECM rework is the reflection of real disease activity and might be an outcome of the structural tissue changes [26] as described in Table 1. There is also involvement of the intestinal stromal cells (myofibroblasts and other mesenchymal populations) in ECM remodeling through the synthesis and reconstitution of the collagen and non-collagenous matrix molecules. The inflamed intestinal mucosa stromal cells release pro-inflammatory chemokines and alter ECM composition, which further encourages tissue remodeling and fibrosis particularly in more complex IBD phenotypes associated with strictures and fibrotic disease [27].

#### 2.1.3. Microbiota Dysbiosis on Extra-Extracellular Matrix and Tissue Structure

**Figure 2:**
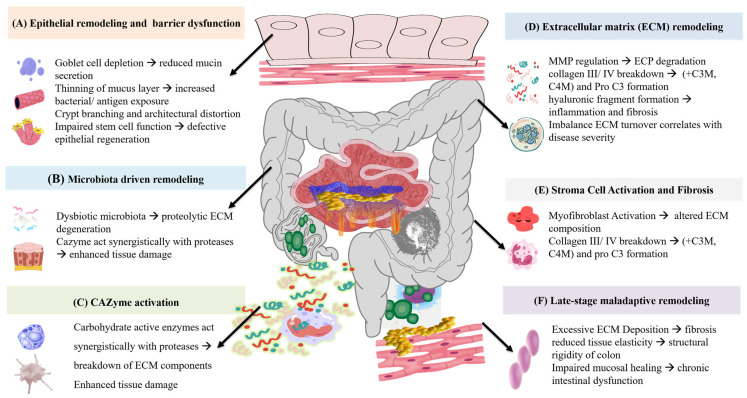
Multi-compartment remodeling in colitis stages driven by host–microbiota interactions, spanning from early epithelial dysfunction to late-stage fibrosis. (**A**) Epithelial remodeling and barrier dysfunction represents the initial stage, characterized by disrupted tight junctions, altered mucin production, and increased intestinal permeability. This barrier compromise facilitates microbial translocation and antigen exposure, leading to (**B**) microbiota-driven remodeling, wherein gut microbiota compositional shifts and metabolic alterations further perpetuate inflammation. Subsequently, (**C**) CAZyme (carbohydrate-active enzyme) activation occurs, primarily derived from specific commensal or pathogenic bacteria, enabling the degradation of dietary fibers and host glycans, which in turn generates metabolites that modulate immune and stromal responses. These events contribute to (**D**) extracellular matrix (ECM) remodeling, involving dysregulated deposition and degradation of collagen, elastin, and other matrix components. Persistent ECM changes then drive (**E**) stroma cell activation and fibrosis, characterized by myofibroblast differentiation, excessive collagen deposition, and progressive tissue stiffening. Finally, (**F**) late-stage maladaptive remodeling ensues, marked by irreversible architectural distortion, stricture formation, and loss of physiological function.

As previously observed, there is also the role of gut microbiota in ECM remodeling. Certain intestinal microbes may feed on proteins and carbohydrates active enzymes (CAZymes), which may directly degrade the ECM components. The bacterial proteolytic activity of stool samples in patients with UC is larger compared to healthy controls, and the supernatants of the ECM-degrading bacteria possess a strong influence regarding the inflammatory reaction in the dextran sodium sulfate (DSS)-induced colitis models. It means that there could be a synergistic outcome of ECM microbial degradation coupled with host proteases in progressive tissue degradation and inflammation [19] as explained in Figure 2.

However, in a later stage of chronic disease it is also possible that over-deposition of the ECM is a factor of fibrosis and structural rigidity of the colon. Although the initial remodeling process implies proteolytic destruction and ECM disappearance, subsequent maladaptive responses to remodeling can lead to adverse deposition of the matrix to disrupt the normal tissue elasticity and functionality [17].

## 3. Dietary Polysaccharides

Carbohydrates consisting of more than 10 molecules of monosaccharide units connected using glycosidic bonds are known as polysaccharides and can be found in numerous living organisms including plants, animals, microbes, fungi, and marine algae. These are grouped into homopolysaccharides, which have one type of monosaccharide in them, e.g., starch, and heteropolysaccharides, which contain more than one type of monosaccharide, e.g., pectin [30]. These polysaccharides act as energy storage compounds and structural building blocks, which are essential for various physiological processes in living organisms [31,32,33].

Natural polysaccharides have tremendous benefits in the treatment of IBD, since they reduce the side effects of the drugs, as well as enhance the effects of therapeutic intervention. Dietary polysaccharides facilitate gut health via enhancing the growth of beneficial microbes, repairing the mucosal intestinal barrier, and promoting the production of SCFA, which alleviates mucosal injury and oxidative stress. As well, polysaccharides can improve barrier resistance to IBD, improve gut microenvironment, and benefit metabolic processes [13,34,35]. Moreover, nanoparticles based on polysaccharides have gained interest due to their capacity to enhance the stability of drugs as well as their release of therapeutic effects [36]. Polysaccharides offer a major hope in the treatment of IBD, considering their dual roles in controlling the gut microbiota and strengthening the intestinal barrier. From a structural perspective, diversity arising from differences in monosaccharide composition, polymerization degree, and glycosidic bonding makes the extraction and purification of polysaccharides a complex process [37]. Their physicochemical characteristics such as composition of monosaccharides, chain lengths, level of branching, and substituents have a significant impact on their bioactivities [38].

Depending on their source, polysaccharides can be categorized as animal, plant, microbial, and marine-derived [39]. Muco-polysaccharides with high water solubility are usually from prior animal sources and are used as candidates in drug development [4]. Plant-derived polysaccharides, including pectin, Angelica, lipopolysaccharide binding protein (LBP-protein), rhubarb, and Bupleurum polysaccharide, are typically soluble in water [40]. Starch and cellulose, on the other hand, are insoluble plant polysaccharides. Bacteria and fungi require the production of microbial polysaccharides, whereas polysaccharides found in aquatic organisms, which are isolated by marine polysaccharides, tend to have specific biological activities [41]. The structural properties of the polysaccharides, like composition of monosaccharides, branching degree, linkage type, and molecular weight, have direct impacts on the relationship between the polysaccharides and the gut microbes with consequent health effects. They influence microbial accessibility and fermentation pathways upon which microorganisms act, ultimately influencing the character and amounts of microbial metabolites generated within the colon, such as SCFA [42].

### Bioactivities of Dietary Polysaccharides Relevant to Colitis and GI Tract Remodeling

Dietary polysaccharides possess a broad spectrum of bioactivities that overlap GI remodeling pathways, which include controlled gut microbiota, enhanced intestinal epithelial barrier, immunomodulation and inhibition of inflammatory signaling. Polysaccharides also impact GI health in colitis through production of SCFA via fermentation process and are also critical as they suppress pathogens, improve nutrient absorption, and increase immune responses in addition to strengthening the mucus barrier [43]. They also reduce intestinal pH, which favors the beneficial bacteria such as *Lactobacillus* and *Bifidobacterium*, which also generate SCFAs and enhance mucosal immunity. Beneficial bacteria are useful in affecting inflammation by balancing the signaling of NF-κB and adhering of epithelial cells. Bacteroidetes and Firmicutes phyla of the gut are known to produce major SCFAs such as acetate, propionate and butyrate. Butyrate, the fats that provide energy and development of intestinal epithelial cells, helps in repairing the mucosa [44].

Recent investigations have shown that selected polysaccharide fraction supplementation has major effects on microbiota composition. Using kiwifruits as an example, polysaccharides reduced the symptoms of DSS-induced UC, increased the concentrations of SCFA in the cecum, and controlled the fucosylation of the colon colonies dependent on microbiota, which induced tight junction proteins expression and induced fucosylation [45]. Alfalfa polysaccharide treatment in colitic mice improved DSS-induced intestinal damage, increased the number of goblet cells, promoted the abundance of beneficial taxa such as *Lachnospiraceae* and *Parabacteroides distasonis*, and reduced the expression of pro-inflammatory cytokines through the TLR4/MyD88/NF-κB signaling pathway [46]. Similarly, polysaccharides from marine jellyfish (*Rhopilema esculentum*) were used in relieving colitis, which suppressed inflammatory levels by rehabilitating the SCFA-producing genera (*Roseburia*, *Bifidobacterium*) as well as replenishing the levels of the SCFAs, which were also linked to the reduction in inflammation and restoration of the functions of the barriers [47].

**Figure 3:**
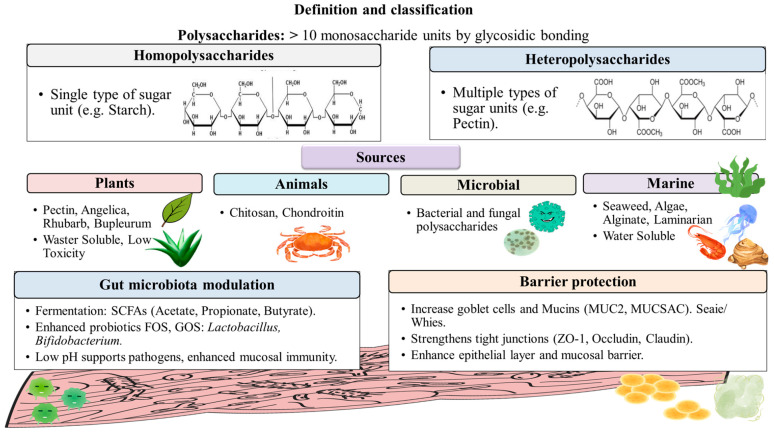
Classification, sources, and gut barrier-related functions of dietary polysaccharides. Definition and classification distinguish two major categories: homopolysaccharides, which consist of a single type of monosaccharide unit (e.g., starch), and heteropolysaccharides, composed of multiple types of sugar units (e.g., pectin). Sources of polysaccharides can be derived from plants, animals, microbial sources, or marine organisms. Gut microbiota modulation: these compounds undergo fermentation to produce short-chain fatty acids (SCFAs), including acetate, propionate, and butyrate. Additionally, prebiotic fibers such as fructooligosaccharides (FOS) and galactooligosaccharides (GOS) enhance the abundance of beneficial genera, particularly Lactobacillus and Bifidobacterium. Fermentation also lowers luminal pH, which can suppress pathogenic bacteria while supporting mucosal immunity. With respect to barrier protection, dietary polysaccharides increase the number of goblet cells and the production of mucins (specifically MUC2 and MUC5AC), strengthen tight junction complexes (including ZO-1, occludin, and claudin), and enhance the integrity of the epithelial layer and overall mucosal barrier. Structures adapted from [53]. Copyright 2026, *Natural Product Communications*.

Mucin production can also be increased through the use of beneficial bacteria supplementation. As an example, intestinal epithelial cells (IECs) express Mucin 2 via lymphocytic-associated pathways, whereas rod-shaped bacteria colonization is sufficient to restore mucus [48]. These results highlight the effects of beneficial bacteria on the health and integrity of the mucus barrier and intestines [49]. Furthermore, SCFAs, which are key metabolites of the gut microbiota, modulate mucus dynamics, stimulating mucin secretion at low concentrations but potentially inhibiting it at high levels. For instance, Alfalfa polysaccharides helped to preserve the intestinal integrity by decreasing the expression of the mucins MUC2 and MUC5AC and increasing the levels of ZO-1, occludin and claudin-1 proteins, and kiwifruit polysaccharides helped to repair the intestinal barrier with the help of interleukin-22 production and the expression of tight junction proteins (ZO-1, Occludin and Claudin-3) in UC mice [45] as illustrated in Figure 3.

The immunomodulatory responses of polysaccharides may involve suppressing pro-inflammatory pathways and cytokines in the pathological pathways that cause colitis. Macrophage activity is also developed by microbial metabolites; in particular, SCFAs suppress the production of pro-inflammatory cytokines by inhibiting the histone deacetylases, whereas anti-inflammatory IL-10 is upregulated by SCFAs [50]. Butyrate specifically suppresses HDAC3, suppresses mTOR-activation and glycolysis, improves macrophage bactericidal activity, and induces an anti-inflammatory phenotype [51]. As an example, *Faecalibacterium prausnitzii* has anti-inflammatory effects, demonstrated by decreasing IL-12 and IFN-α, increasing IL-10 secretions, and blocking NF-κB signatures [52]. Overall, these studies show that dietary polysaccharides can regulate microbial metabolism, improve epithelial repair and ameliorate immune signaling, alter matrix processes, and help in modulating gastrointestinal remodeling reactions during colitis, as well as have potential nutritional effects of restoring intestinal structure and intestinal function in IBD. This suggests that dietary polysaccharides have potential nutritional effects such as restoring intestinal structure and intestinal functioning in cases of inflammatory bowel diseases.

## 4. Structural Characteristics of Plant-Derived Active Polysaccharides

The structural characterization of polysaccharides reveals a profound correlation between their molecular architecture, specifically monosaccharide composition, glycosidic linkage types, branching patterns, and molecular weight, and their biological activities in colitis models [54,55]. However, a critical examination of the literature reveals that few universal rules exist and many claimed structure–activity relationships are source-specific or confounded by extraction methods, purity, and experimental models. This section synthesizes the available data to identify consistent patterns where they exist, while explicitly acknowledging contradictions and knowledge gaps.

### 4.1. Molecular Weight: A Non-Linear and Source-Dependent Determinant

Perhaps the most frequently cited structural parameter is molecular weight, yet the relationship between MW and bioactivity is neither linear nor universal. The most detailed evidence comes from *Astragalus membranaceus* polysaccharides, which serve as a paradigm for structure–activity relationships, where fractionation by MW yields distinct bioactive profiles. APS fractions with molecular MW > 2000 kDa, approximately 10 kDa, and about 300 Da exhibit differential immune activities, with no single fraction being uniformly superior across all outcomes [56]. Critical observation described that low molecular weight (2.60–8.38 KDa) alleviates dextran sulfate sodium (DSS)-induced colitis in mice by restoring short-chain fatty acid production and regulating Th17/Treg cell homeostasis in a microbiota-dependent manner [12]. However, the same study notes that further degradation below 2 kDa substantially reduces activity, suggesting an optimal MW window rather than a simple “lower is better” rule. This pattern is not universal. *Hedysarum* polysaccharides (HG-2, -3, -4) show a decreasing degree of branching with decreasing molecular weight, suggesting that structural compactness may influence their biological accessibility [56]. In contrast, for *Astragalus arabinogalactans*, the high MW fraction APS2-I (1.96 × 10^6^ Da) and APS3-I (3.91 × 10^6^ Da) exhibits the strongest antioxidant and cardio-protective activities [57].

These contradictory findings indicate that optimal MW is polysaccharide-specific, determined by source, monosaccharide composition, and backbone structure. No universal “optimal MW” exists.

### 4.2. Degree of Branching

Degree of branching (DB) is frequently invoked as a determinant of bioactivity, but the relationship appears non-linear and context-dependent. High DB is generally associated with enhanced microbial accessibility and prebiotic effects, presumably because branched structures provide more terminal sugar residues for microbial enzymes. Black mulberry polysaccharide (DB 0.38), kiwifruit polysaccharide (DB 0.32), and noni fruit polysaccharide (DB 0.41) all show strong prebiotic activity [58,59]. However, high DB may reduce direct TLR binding by sterically hindering access to backbone recognition motifs. For *Hedysarum* polysaccharides, DB decreased systematically from HG-2 (highest DB) to HG-4 (lowest DB), yet HG-4 showed the strongest anti-inflammatory activity, suggesting that excessive branching may impede bioactivity in certain contexts [56]. Conversely, for type II arabinogalactans from *Astragalus*, higher DB correlates with enhanced immunomodulatory activity [60].

### 4.3. Monosaccharide Composition: Patterns and Exceptions

Certain compositional patterns emerge consistently. Polysaccharides rich in galactose and rhamnose, such as *Lycium barbarum* polysaccharide, exhibit high uronic acid content and sustained fermentation profiles, indicative of strong prebiotic functionality [58]. Pectic domains rich in galacturonic acid (GalA) often serve as recognition sites for immune receptors [61]. The exceptions abound. Chinese yam polysaccharide combines starch-like α-1,4 glucose linkages with pectin domains, yet its anti-inflammatory activity is entirely microbiota-dependent; it shows no effect in germ-free mice [62]. This suggests that monosaccharide composition alone cannot predict the mechanism; the same sugars in different linkage contexts produce different outcomes. Similarly, buckwheat polysaccharides FTP (β-1,3/β-1,6 glucan) and FEP (pectic) coexist in the same plant but have distinct effects: FTP excels at immunomodulation, while FEP is superior for barrier protection [58].

Monosaccharide composition provides a necessary but insufficient predictor of bioactivity. Linkage type, branching pattern, and molecular weight must be considered together. The field lacks systematic studies that vary one structural parameter while holding others constant.

### 4.4. Chemical Modifications

Chemical modification is a promising strategy for enhancing or tuning polysaccharide bioactivity, but the field lacks standardization. Sulfation and acetylation consistently enhance or redirect polysaccharide bioactivity, but the degree and position of substitution are critical. Sulfation of mulberry leaf polysaccharides at O-2 and O-6 positions (degree of substitution DS 0.42) preserves the native backbone while introducing negative charges that enhance biological activities [63]. Acetylation of *Polygonatum sibiricum* fructooligosaccharide at the O-6 position (DS 0.15) significantly enhances immunoactivity by activating Dectin-1 and CD14 receptors [64]. The literature contains no systematic dose–response studies for DS. It is unknown whether DS 0.42 is optimal or whether higher DS would increase efficacy or toxicity. Most modified polysaccharides are compared only to their native forms, not to each other or to positive controls, making it impossible to determine which modification strategy is superior. Furthermore, chemical modification introduces batch-to-batch variability and raises regulatory concerns. The comprehensive analysis of these structural features provides a robust foundation for understanding the mechanism of action of polysaccharides in Table 2.

## 5. Gastrointestinal Tract Remodeling Induced by Polysaccharides

Dietary polysaccharides induce gut remodeling via a bunch of activities and mechanisms, including restoration of mucosal architecture, histopathological improvements, enhanced gut motility and anti-fibrotic remodeling. The composition and functions of gut are interrupted by the collective properties of prebiotics, antioxidants and anti-inflammatory effects of polysaccharides [2,71]. Further, we discuss the differences between studies regarding their respective models and their effectiveness.

### 5.1. Hierarchy of Evidence: Comparing Model Systems for Polysaccharide Research in Colitis

The evidence presented in this review is derived from distinct experimental systems, each with fundamentally different translational strengths. Understanding this hierarchy is essential for correctly interpreting the mechanistic factors such as the following: animal models provide proof-of-concept efficacy data, cell culture studies elucidate direct epithelial mechanisms, and fermentation studies reveal microbial metabolic pathways; no human clinical trial data are currently available as described below. The key mechanistic actions are sourced from evidence grading, models used across different studies, and their respective limitation of dietary polysaccharides in colitis, as listed in Table 3.

#### 5.1.1. Animal Model Studies

In a murine model of dextran sulfate sodium-induced colitis, treatment with various natural polysaccharides has consistently demonstrated significant therapeutic efficacy. Polysaccharides from *Grifola frondosa* and *Inonotus obliquus* administered at 200 mg/kg/day by gavage markedly ameliorated colitis symptoms, reduced disease activity index (DAI), prevented colon shortening, and modulated both systemic metabolism and gut microbiota composition [72]. Similarly, tamarillo fruit pulp polysaccharides, specifically type I arabinogalactan and methyl-esterified homogalacturonan, showed protective effects against DSS-induced UC by reducing inflammation and promoting mucosal healing [73]. Epimedium polysaccharide treatment mitigated UC symptoms including weight loss, anal bleeding, elevated DAI, and colon shortening, while also inhibiting oxidative stress and regulating autophagy pathways [74]. Chinese yam polysaccharide (YP) purified from *Dioscorea opposita Thunb* showed therapeutic efficacy in UC mice, complemented by in vitro studies which demonstrated significant attenuation of inflammatory responses in LPS-stimulated intestinal epithelial cells [62]. Polysaccharides from natural *Cordyceps sinensis* attenuated DSS-induced colitis in C57BL/6J mice through histological, serological, biochemical, and immunologic improvements [75]. An acidic polysaccharide from *Phellinus linteus* demonstrated ameliorative effects on UC in a DSS-induced mouse model [76]. Glycyrrhiza pallidiflora polysaccharide (GPP) ameliorated DSS-induced colitis by protecting intestinal barrier integrity across multiple dose levels (100, 200, 300 mg/kg) [77].

Crude polysaccharides from *Physalis pubescens L.* mitigated colitis by preventing oxidative damage, aberrant immune responses, and dysbacteriosis [78]. Rhamnogalacturonan (RGal) from *Acmella oleracea* leaves improved intestinal barrier function in DSS-induced colitis mice [79]. Homogalacturonan from yellow passion fruit peel ameliorated intestinal injury in DSS-induced colitis mice [80]. Polysaccharide from *Millettia speciosa* Champ roots showed protective effects in DSS-induced UC mice [81]. Stropharia rugosoannulata polysaccharide (SP-1a) and its derivatives alleviated weight loss, colon shortening, and bloody stools induced by DSS [82].

Natural plant polysaccharides generally alleviate DSS-induced UC by inhibiting inflammation and modulating gut microbiota. Turmeric polysaccharides ameliorated DSS-induced UC by modulating gut microbiota, improving microbial metabolites, and enhancing gut barrier function [83]. Glucan from *Gastrodia elata Blume* showed ameliorative effects on DSS-induced colitis in mice [84]. Bergamot polysaccharides relieved DSS-induced UC via regulating gut microbiota and metabolites [85]. Rhopilema esculentum polysaccharides enhanced epithelial cell barrier in vitro and alleviated chronic colitis in mice [86]. Exopolysaccharides from *Limosilactobacillus mucosae* CCFM1273 alleviated UC via modulation of gut microbiota and inhibition of Fas/Fasl and TLR4/NF-κB pathways [87].

The high-impact study on Eucommia ulmoides polysaccharide-modified nano-selenium (EUP-SeNP) revealed that this formulation effectively alleviated DSS-induced colitis by enhancing intestinal mucosal barrier function and antioxidant capacity, demonstrating the potential of polysaccharide-based nanotherapeutics [88]. Degraded polysaccharides from *Sargassum fusiforme* (PSF-T2) prepared by UV/H_2_O_2_ treatment effectively ameliorated DSS-induced colitis [89]. The special polysaccharide hydrogel from *Brasenia schreberi* (BS mucilage) showed preventive effects against UC, even at very low solid content (0.3%), through modulating gut microbiota [90]. Agaricus blazei Murrill polysaccharide protected against DSS-induced colitis by modulating intestinal barrier function and remodeling metabolism [89].

**Figure 4:**
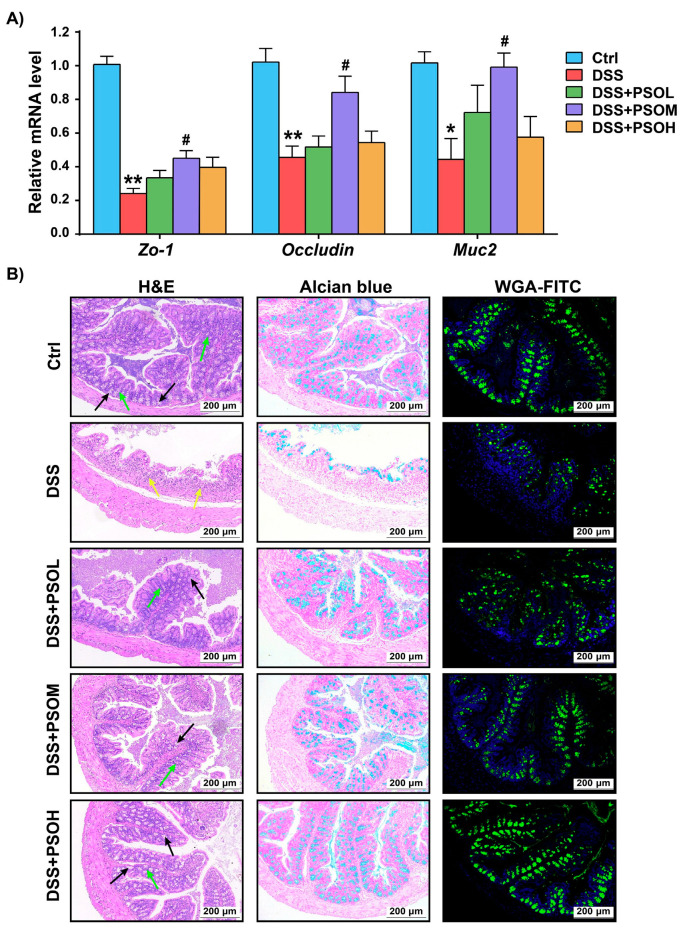
Preparation, structural characterization, and colitis-ameliorating effects of a natural acetylated fructooligosaccharide (PSO-A) derived from Polygonatum sibiricum, mediated via NLRP3 inhibition. PSO-A alleviates intestinal mucosal injury in DSS-induced colitis mice. (**A**) Relative mRNA expression levels of *Zo-1*, Occludin, and Muc2 in colonic tissues, determined by qRT-PCR. Data are presented as mean ± SEM (n = 8). * *p* < 0.05, ** *p* < 0.01 versus control (Ctrl) group; # *p* < 0.05 versus DSS-treated group. (**B**) Representative histological and histochemical images of colon tissues following hematoxylin and eosin (H&E) staining, Alcian blue staining, and WGA-FITC staining (all at 100× magnification). Black arrows indicate goblet cells; green arrows denote crypts; yellow arrows point to inflammatory cell infiltration. The pathway schematic is adapted from [93] copyright 2026, *Journal of Functional Foods*/Elsevier.

Similarly, *Selaginella uncinate* (SUSP-4), being the rich source of polysaccharides, prevents or inhibits macrophage activation. Colonic lining is restored, and *Akkermansia muciniphila*, a beneficial bacteria, is enhanced in IBD mice to restore intestinal mucosal status [91]. Monosaccharide composition, molecular weight and glycosidic linkages contribute to the therapeutic efficiency of polysaccharides [13,92]. As shown in Figure 4, the acetylated fructooligosaccharide PSO-A from *Polygonatum sibiricum* (molecular weight 2.12 kDa) hinders the NLRP3 inflammasome pathway and mitigates the colitibers in DSS-induced mice [93].

*Artocarpus heterophyllus Lam.* (Jackfruit) has significant polysaccharides in the pulp. It improves the intestinal mucosal barrier via inhibiting oxidative stress in high-fat-diet-induced rats. Its application also reduces inflammation [94]. In a nutshell, polysaccharides have been proved crucial in gut homeostasis through intestinal mucosal architecture and gut barrier enhancement.

Polysaccharides show properties that regulate cellular pathways, contributing to fibrosis. Tissue repair and immunomodulation can also be achieved by the polysaccharides obtained from *Albuca bracteate*. The *A. bracteate*-derived polysaccharides diminish colon tumorigenesis through the administrative control of inflammation, gut microbiota and oxidative stress in AOM/DSS-induced mice [95]. Inflammation and immunity regulation by polysaccharides make them a promising complementary strategy due to their anti-fibrosis activity [71]. This immunity and inflammation regulation also helps in the inhibition and control of fibrotic remodeling, building intestinal epithelial barrier strength [96].

The enteric nervous system and muscularis externa are two main structures that maintain gut motility. Polysaccharides upgrade gut motility, distinguished from anti-inflammatory activity. Polysaccharides obtained from *Hericium erinaceus* are able to reduce colon structural damage and DAI in antibiotic-associated diarrhea (AAD) rats. This shows the ability of polysaccharides to improve gut integrity [97]. Gut dysmotility causes diarrhea, which can be treated by polysaccharide application. *Panax quinquefolius* polysaccharides (WQP) application assists in enhanced gut activity via regulating the gut microbiota and signaling pathways in AAD rats [98]. Gut motility is also affected positively by SCFA production, e.g., butyrate helps to maintain the gut by facilitating colonocyte health improvement and intestinal barrier strength. For instance, a crucial prebiotic, inulin, is a polysaccharide that treats intestine destruction via PI3K/AKT/mTOR signaling pathway regulation [99,100]. In short, the enteric nervous system and muscularis externa are concerned with the whole of gut motility and health improvement, with specific functions for growth and metabolism.

**Figure 5:**
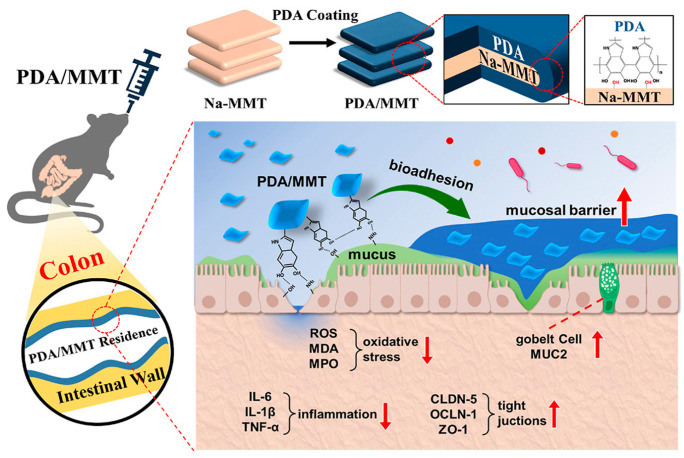
Polydopamine-cladded montmorillonite (PDA/MMT) micro-sheets as a therapeutic platform for repairing the gut mucosal barrier in murine colitis through oxidative stress inhibition. PDA coating of Na-MMT enhances bio-adhesion and colonic residence time. PDA/MMT micro-sheets reduce oxidative stress (ROS, MDA, MPO), protect goblet cells and MUC2, restore tight junction proteins (ZO-1, OCLN-1, CLDN-5), and suppress pro-inflammatory cytokines (IL-6, IL-1β, TNF-α), leading to mucosal barrier repair and colitis alleviation; adapted from [101] copy right 2026, *Materials Today Bio*, Elsevier.

Another way to treat gut mucosal barrier malfunction is polydopamine-cladded montmorillonite micro-sheets (PDA/MMT). These sheets show therapeutic activity for colitis via oxidative stress inhibition [101], as shown in Figure 5. In conclusion, polysaccharides have functional and therapeutic roles in the cure for GIT disorder. They can build up gut motility, gut remodeling, and microbiota regulation. The anti-inflammatory effects, along with anti-fibrotic effects, barrier-enhancing impact, and restoration of gut motility, lead histopathological betterment and mucosal architecture restoration of the gut [2,71].

In summary, numerous previous murine studies have demonstrated that diverse polysaccharides from fungal, plant, and marine sources consistently alleviate DSS-induced colitis symptoms, reduce DAI, restore colon length, and improve histological outcomes. The consistency of these findings across multiple laboratories and polysaccharide sources provides strong proof-of-concept evidence that dietary polysaccharides possess genuine anti-colitis activity.

#### 5.1.2. Cell Model Studies

Using a Caco-2 cell monolayer, it was demonstrated that several polysaccharides directly enhance epithelial barrier integrity. Specifically, exopolysaccharide produced by *Streptococcus thermophilus* showed alleviating effects on intestinal inflammation and mucosal barrier function in Caco-2 monolayers [102]. Rhamnogalacturonan isolated from *Acmella oleracea* leaves demonstrated protective and healing effects on heterogeneous human epithelial colorectal adenocarcinoma cells, improving intestinal barrier function [79]. *Astragalus* polysaccharides directly upregulate tight junction proteins in Caco-2 monolayers [103]. *Rhopilema esculentum* polysaccharides enhanced epithelial cell barrier function in vitro as measured by Caco-2 cell monolayer assays [86]. These studies typically measure outcomes such as transepithelial electrical resistance and FITC-dextran flux to quantify barrier integrity improvements without cytotoxicity at tested concentration ranges.

In summary, studies using Caco-2 cell monolayers have demonstrated that specific polysaccharides directly enhance epithelial barrier integrity, as measured by increased transepithelial electrical resistance (TEER), reduced FITC-dextran flux, and upregulation of tight junction proteins (ZO-1, occludin, claudin). These findings establish mechanistic plausibility for microbiota-independent, direct epithelial effects of polysaccharides. However, the translational strength of this evidence is low to moderate. Caco-2 monolayers are a simplified, single-cell-type system that lacks immune cells, gut microbiota, enteric nervous system functions, and the complex three-dimensional crypt villus architecture of the intestinal mucosa.

#### 5.1.3. Fermentation-Based Studies

In vitro fermentation with human fecal inoculum showed that certain polysaccharides are selectively metabolized by the gut microbiota to produce beneficial SCFAs. Comprehensive evaluation of *Flammulina velutipes* residue polysaccharide demonstrated its fermentation characteristics using human fecal inoculum under controlled anaerobic conditions [102]. Similarly, intrapolysaccharide from *Paecilomyces cicadae* underwent artificial simulated digestion followed by in vitro fermentation with human gut microbiota, revealing its gastrointestinal metabolism characteristics [104]. Basidiospore-derived exopolysaccharides from *Naematelia aurantialba* were also evaluated for in vitro digestion and fecal fermentation with human gut microbiota [105]. These fermentation studies typically show selective metabolism to SCFAs and shift in microbial composition toward saccharolytic taxa relative to control fibers.

Most polysaccharides reach the colon undigested and exert effects primarily through fermentation-dependent (indirect) mechanisms, though some sulfated or low-MW fractions may directly interact with epithelial TLRs. Polysaccharides can directly bind to epithelial TLR2 and TLR4, modulating NF-κB signaling without microbial involvement [31,106]. Germ-free mice fed with polysaccharides fail to produce SCFAs and show no anti-colitis activity, suggesting indirect effects on the gut [107]. Polysaccharides indirectly affect SCFA production, secondary bile acid metabolism, and tryptophan metabolite generation, which are completely microbiota-dependent [31,108,109].

Studies using human fecal inoculum under anaerobic conditions have demonstrated that specific polysaccharides are selectively fermented by the gut microbiota to produce short-chain fatty acids (SCFAs), particularly acetate, propionate, and butyrate, while shifting microbial composition toward saccharolytic taxa. These findings provide valuable insights into how polysaccharides are metabolized by human-derived gut microbes and support the microbiota-dependent mechanism of action.

#### 5.1.4. Human Clinical Studies

To date, no human randomized controlled trials have been published on any specific polysaccharide for ulcerative colitis. The available evidence remains entirely preclinical, limited to murine models of DSS-induced colitis, in vitro cell culture systems (primarily Caco-2 monolayers), and in vitro fermentation studies with human fecal inoculum. While these preclinical studies provide compelling mechanistic insights and demonstrate consistent therapeutic potential across diverse polysaccharide sources, the absence of human clinical trial data represents a critical translational gap. This lack of clinical evidence underscores the urgent need for well-powered, placebo-controlled, randomized controlled trials to validate the safety and efficacy of these promising natural compounds in human UC patients before they can be considered viable therapeutic alternatives to conventional treatment.

**Table 3:** Level of studies for key mechanistic actions of dietary polysaccharides in colitis.

Mechanism	Polysaccharide Sources	Evidence Grading	Primary Model	Consistency Across Studies	Key Limitations	Reference
Enrichment of *Lactobacillus* and *Bifidobacterium*	*Astragalus*, *Chrysanthemum*, mulberry, kiwifruit, *Ganoderma*, *Pleurotus*, inulin, FOS, GOS	★★★★★ Strong	Animal models (DSS mice)	Highly consistent across >15 independent studies; independent of MW and DB	Murine model only; no human validation	[8,9,10,43,45,60,110,111,112,113,114,115,116]
Enrichment of *Akkermansia muciniphila*	Sulfated mulberry leaf polysaccharide (SMLP), *Rhopilema esculentum*, *Gracilaria lemaneiformis*, *Selaginella uncinata* (SUSP-4)	★★★☆☆ Moderate	Animal models (DSS mice)	Condition-dependent; consistently observed with sulfated and marine-derived polysaccharides	Requires specific polysaccharide structures; not universal	[13,63,70,86,92,110,111]
Suppression of *Enterobacteriaceae*	*Chrysanthemum morifolium*, *Pleurotus eryngii*, mulberry	★★★★☆ Strong	Animal models and vitro fermentation	Consistent across multiple studies	Species-specific effects may vary	[58,110,111,117,118]
SCFA production	*Aloe vera*, *Meconopsis*, *Ganoderma lucidum*, almond, *Flammulina velutipes*, *Paecilomyces cicadae*, *Naematelia aurantialba*	★★★★☆ Strong	Animal models and in vitro fermentation (human fecal inoculum)	Consistently demonstrated across ≥20 animal studies and ≥4 fermentation studies	Fermentation studies lack host response; animal models show increased SCFAs but human data lacking	[102,104,105,112,113,114,119]
GPCR activation	SCFAs (butyrate, propionate, acetate)	★★★★☆ Strong	Animal models and in vitro	Well-established signaling pathway; consistent across models	Derived from SCFA studies, not direct polysaccharide-receptor binding	[110,111,120,121,122,123]
HDAC inhibition	Butyrate, propionate	★★★★☆ Strong	Animal models and in vitro	Well-established mechanism; consistent across multiple studies	Mechanism inferred from SCFA effects	[2,5,122,123,124]
Tight junction upregulation	*Astragalus*, *Scutellaria baicalensis*, noni fruit, *Tremella fuciformis*, *Rhopilema esculentum*, *Gracilaria lemaneiformis*	★★★★★ Strong	Animal models and Caco-2 cell culture	Highly consistent across ≥15 animal studies and ≥5 cell culture studies	Cell culture lacks systemic complexity; human data absent	[12,60,65,66,86,110,111,117]
Necroptosis inhibition	*Lentinus edodes* (shiitake) polysaccharides	★★☆☆☆ Limited	Caco-2 cell culture and animal models	Emerging mechanism; limited studies	Few polysaccharides tested	[103]
NF-κB signaling suppression	*Astragalus membranaceus*, *Chrysanthemum morifolium*, *Scutellaria baicalensis*, *Eucheuma cottonii*, *Sagittaria sagittifolia*, *Limosilactobacillus mucosae*	★★★★★ Strong	Animal models and macrophage cell culture	Highly consistent across ≥15 independent studies	No human clinical validation	[12,58,60,65,87,117,125,126]
MAPK signaling suppression	*Ganoderma lucidum*, *Ganoderma sinense*, *Pleurotus eryngii*, *Sagittaria sagittifolia*, *Dictyophora indusiata*	★★★★☆ Strong	Animal models and macrophage cell culture	Consistent across multiple studies	Fewer studies than NF-κB; mostly in macrophage models	[35,106,113,118,126,127]
NLRP3 inflammasome inhibition	*Polygonatum sibiricum* PSO-A (acetylated fructooligosaccharide), *Scutellaria baicalensis*	★★★★☆ Strong	Animal models (DSS mice)	Well-documented for specific polysaccharides	Polysaccharide-specific; not universal	[66,67,68,117]
Th17/Treg balance restoration	*Astragalus membranaceus*, *Atractylodes macrocephala*, *Poria cocos*, Huangshan Floral Mushroom	★★★★☆ Strong	Animal models	Consistent across multiple studies	Primarily in murine models; human translation unclear	[12,60,65,128,129]
M2 macrophage polarization	Butyrate, *Aloe vera*, *Meconopsis*	★★★★☆ Strong	Animal models and macrophage cell culture	Well-established for butyrate; emerging for polysaccharides	Direct polysaccharide evidence limited	[114,119,120,130]
Treg induction	SCFAs (butyrate), *Astragalus membranaceus*, *Tremella fuciformis*	★★★★☆ Strong	Animal models	Consistent across multiple studies	Mechanism primarily via SCFAs	[12,60,65,131]
Nrf2/HO-1 antioxidant pathway activation	*Aloe vera*, *Hericium erinaceus*, *Lycium barbarum*	★★★★☆ Strong	Animal models and cell culture	Consistent across multiple studies	Limited number of polysaccharides tested	[97,114,120,130,132]
Oxidative stress reduction	*Epimedium*, *Gracilaria caudata*, black mulberry, *Hericium erinaceus*, *Physalis pubescens*	★★★★★ Strong	Animal models and cell culture	Highly consistent across ≥10 independent studies	No human clinical validation	[74,91,97,114,132,133]
Goblet cell restoration and mucin production	Alfalfa, kiwifruit, *Tremella fuciformis*	★★★★☆ Strong	Animal models	Consistent across multiple studies	Primarily histological evidence	[45,46,131]
Extracellular matrix remodeling	*Albuca bracteata*, general polysaccharides	★★☆☆☆ Limited	Animal models (AOM/DSS)	Emerging area; limited direct evidence	Conceptually proposed but mechanistically understudied	[95]
Gut motility improvement	*Hericium erinaceus*, *Panax quinquefolius*, inulin	★★★☆☆ Moderate	Animal models (AAD rats)	Consistent across several studies	Primarily in diarrhea models; not colitis-specific	[97,98,99,100,132]
Anti-fibrotic remodeling	*Albuca bracteata*, general polysaccharides	★★★☆☆ Moderate	Animal models	Emerging area; limited direct evidence	Inferred from anti-inflammatory effects	[87,95,126]

This table summarizes the current evidence level for each mechanistic action of dietary polysaccharides in colitis based on available cellular, fermentative, animal, and human studies. Evidence strength is graded using a standardized five‑star system: ☆☆☆☆☆, no supportive evidence; ★☆☆☆☆, limited preliminary evidence from in vitro or fermentative models; ★★☆☆☆, consistent evidence from multiple in vitro/fermentative studies or preliminary in vivo animal data; ★★★☆☆, solid evidence from consistent animal model studies with partial mechanistic validation; ★★★★★, robust and conclusive evidence supported by repeated in vivo verification and/or human clinical data.

## 6. Mechanistic Actions of Polysaccharides in Colitis

### 6.1. Microbiota-Dependent Mechanisms

#### 6.1.1. Gut Microbiota Remodeling

UC is characterized by reduced microbial diversity, primarily by a reduction in Bacteroidetes, *Eubacterium rectale* and *Akkermansia muciniphila* alongside increased abundance of potentially pathogenic *Enterobacteriaceae* (particularly *Escherichia coli*) [115,116]. Environmental factors including dietary habits and smoking status may influence an individual to UC susceptibility by modulating gut microbial composition, though the impact of smoking on UC in Asian populations remains unclear [110,111]. However, a critical evaluation of the literature reveals that certain microbial changes are consistently reproducible across diverse polysaccharide sources and experimental models, while others are model-specific or polysaccharide-dependent.

Polysaccharides isolated from *Chrysanthemum morifolium* have demonstrated beneficial effects in the protection against UC. The signal transduction pathways, such as block NF-κB and JAK/STAT, might be blocked by chrysanthemum polysaccharide. By inhibiting these unique cascades, *Chrysanthemum* polysaccharide reduces the expression of some cytokines and proteins [117]. Further, based on 16S rRNA sequencing analysis, in colitis, the gut flora can also become imbalanced. Treatment with this particular polysaccharide maintains an inequitable microbiome as it reduces the prevalence of opportunistic microflora such as *Escherichia* and *Enterococcus* and increases the populations of *Butyricicoccus*, *Clostridium*, *Lactobacillus*, *Rikenellaceae* and *Bifidobacterium*. Moreover, this polysaccharide not only retains the dysregulated gut pathogens but also prevents pro-inflammatory factors such as IL-23, TNF-α, and interferon-gamma, which were the reasons for colonic intestinal damage [117]. *Gracilaria lemaneiformis* oligosaccharides also exert their effects through maintaining the intestinal microbiome and reinforcing the short-chain fatty acids. This polysaccharide modulates the markers in the immune organs and proteins for inflammatory response and the expression of metabolites in the intestine. Structural characteristics of these oligosaccharides were undertaken in alleviation of DSS-induced colitis by modulating gut microbiota and intestinal metabolites [114].

Multiple studies demonstrate that microbial changes are consistently related to *Lactobacillus*, and *Bifidobacterium* represents the most robust and reproducible finding across mice models. Majority of the independent studies have demonstrated consistent increases in relative abundance of these genera following intervention with diverse polysaccharide sources, including plant-derived (*Astragalus*, *Chrysanthemum*, mulberry, kiwifruit, *Sagittaria*, buckwheat, *Aloe*), fungal-derived (*Ganoderma*, *Pleurotus*, *Flammulina*), marine-derived (*Rhopilema*), and prebiotic fibers (inulin, FOS, GOS) [8,9,10,45,86,110,111,112,113]. This effect is independent of molecular weight (2–150 kDa) and degree of branching and has been replicated in both dextran sulfate sodium and 2,4,6-trinitrobenzenesulfonic acid UC-induced mice models [46].

Enrichment of *A. muciniphila* has been reported in previous studies but shows clear condition dependence. Increased abundance is consistently observed with sulfated polysaccharides, such as sulfated mulberry leaf polysaccharide (SMLP), and marine-derived polysaccharides from *Rhopilema esculentum* and *Gracilaria lemaneiformis* [91,114]. Similarly *Butyricicoccus* and *Rikenellaceae* were increased exclusively by *Chrysanthemum morifolium* polysaccharide in a single DSS-induced UC in rat study [117]. *Parabacteroides distasonis* enrichment was reported only with alfalfa polysaccharide [46]. Despite its strong relevance to IBD, *Faecalibacterium prausnitzii* has been investigated in studies focusing on polysaccharides [8,9,10,113]. *Ruminococcaceae* has been reported in *Ganoderm* and buckwheat polysaccharides, suggesting a possible requirement for β-glucan structures [113,134].

Other gut microbiota like *Pleurotus eryngii* resist digestion in the upper gastrointestinal tract and reach the colon intact, where they are fermented by saccharolytic bacteria. This fermentation process enriches specific microbial taxa and alters metabolic output. For instance, in vitro fermentation of *Pleurotus eryngii* mushrooms with human fecal inoculate significantly increased the relative abundance of butyrate-producing bacteria, including members of *Lachnospiraceae* and *Ruminococcaceae*, while reducing pro-inflammatory genera such as *Escherichia-Shigella* [127]. Similarly, administration of protein from *P. eryngii* in murine colitis models led to a notable decrease in *Escherichia* abundance and an increase in beneficial *Lactobacillus* species, correlating with reduced inflammatory cytokines and improved barrier integrity [118].

#### 6.1.2. SCFA Production and Signaling (GPCR and HDAC Pathways)

GI disorders have been revealed to be significantly influenced by metabolites originating from the gut microbiota, such as SCFAs. The most prevalent colonic SCFAs generated by intestinal microbial fermentation of dietary fiber are acetate (C2), propionic (C3), and butyrate (C4). They serve as signal molecules by blocking the action of the histone deacetylase (HDAC) and activating the G-protein-coupled receptors (GPCRs) such as GPR41, GPR43, and GPR109A [120,121]. GPCR activation in turn controls a variety of cellular processes, such as immunology, metabolism, and inflammatory response. Some of the primary metabolites of microbes, SCFAs are produced when bacteria ferment food fiber and are mostly used in the colon as a source of energy by colon cells [135]. Fatty acids with fewer than six carbon atoms are known as SCFAs [136]. They are mostly produced by gut bacteria through the fermentation of resistant starch and undigested fiber, while they can also be produced spontaneously in the liver through host metabolic pathways. Through apoptosis (programmed cell death) and tumor cell growth, SCFAs prevent carcinogenesis [122]. SCFAs, or short-chain fatty acids, have demonstrated significant promise as a therapeutic approach for IBD [137]. Butyrate-producing bacteria and the expression of the receptors GPR43 and GPR109A are both markedly reduced in individuals with colon cancer, demonstrating the preventive impact of SCFAs against colitis and colon cancer [124].

SCFAs increase neutrophils’ phagocytic potential and encourage their migration to an infection site in reaction to inflammation, which aids in the infection’s treatment. Butyrate and propionate are histone deacetylase (HDAC) inhibitors. HDAC inhibition suppresses pro-inflammatory cytokine production (TNF-α, IL-6, IL-1β) in macrophages. Inhibiting HDAC and GPCR signals are the main ways to aid in controlling T-cell function. Additionally, SCFAs have an impact on T-cell development in dendritic cells (DCs). SCFAs also contribute to their development in several subsets, including Th1, Th17, and Tregs, within a complicated cytokine context. By blocking HDC, SCFAs cause B cells to produce intestinal IgA. In the gut, this immunoglobulin is in charge of immunological tolerance [123]. Butyrate ameliorated colonic inflammation in mice by reducing the level of pro-inflammatory cytokines such as interleukin-6, IL-17, TNF-α and IL-1beta [138]. SCFAs participate in modulating immune cells. Butyrate was found to ameliorate colitis in mice due to the inhibition of pro-inflammatory T helper 17 cells by the activation of Sirtuin 1 and mammalian target of rapamycin [138].

Butyrate was revealed to promote M2 (anti-inflammatory) macrophage polarization, which suppressed M1 intestinal inflammation in in vitro and in vivo models of colitis [139] as described in Table 4. Polysaccharides help to alleviate UC, in part, by increasing production of SCFAs important for intestinal barrier function and immunomodulation. For example, *Aloe vera* polysaccharides balance the concentrations of SCFAs and also activate the Nrf2/HO-1 pathway, providing protection to the layers of the intestine and modulating the antioxidant and inflammatory genes [120]. Similarly, *Meconopsis* polysaccharides (MP) restore SCFA concentrations, such as butyrate, propionic acid and valeric acid, while promoting beneficial gut bacteria and reducing harmful bacteria, improving immune regulation and intestinal barrier integrity [119]. *Ganoderma lucidum* polysaccharide (GLP) improves cecal SCFAs through its ability to improve SCFA-producing bacteria such as *Ruminococcus*, which supports epithelial gene expression and immune function [113]. Almond polysaccharide, named AP-1, is also shown to restore SCFA profiles as well as increase goblet cells and inhibit epithelial apoptosis and pro-inflammatory cytokines, modulating both innate and adaptive immune responses [140]. These actions all work in concert to maintain gut homeostasis, inhibit inflammation, and boost the immune defense system in colitis models.

### 6.2. Microbiota-Independent and Direct Epithelial Mechanisms

#### 6.2.1. Tight Junction Regulation

Tight *junctions* (TJs), which are clusters of complex structures formed between neighboring intestinal epithelial cells, are primarily responsible for forming the intestinal mucosal barrier. Although TJ modulation is an efficient way to improve absorption, inflammation often causes the TJ barrier to be disrupted [146]. The transmembrane and cytosolic proteins, occludin, claudin, zonula occludens (ZOs), tricellulin, cingulin, and junctional adhesion molecules (JAM), are connected to the TJs [147]. TJ proteins play a significant role in the pathophysiology of UC, as reported by an increasing number of studies conducted on colitis models [148]. Goblet cells produce and secrete mucus, which covers the intestinal lining’s surface and is crucial to the integrity of the intestinal barrier. This gel-like material serves as a barrier between the intestinal contents and the host’s tissue, providing a home for the gut microbiota and protecting the intestinal lining [149]. Research has indicated that UC patients had higher expression of mucin, a crucial component of mucus [149]. Bacteria and their structures can influence the mucus barrier in a certain manner, which may impact health. A healthy community in the microbiome is crucial to the balance in the homeostasis of the mucosal barrier, which consists of a dynamic balance of production, secretion, expansion and proteolysis of mucus components [115].

Polysaccharides help maintain the integrity of the intestinal epithelium through the regulation of cell death pathways such as apoptosis and necroptosis. For example, polysaccharides block RIPK1-RIPK3-MLKL necroptotic signaling to reduce the phosphorylation levels of MLKL and reduce necroptotic cell death in Caco-2 cells. Their composition of carbohydrates is key to these anti-necroptotic effects [103]. Similarly, *Tremella fuciformis* and other polysaccharides of plant origin exert their protection mechanism through the maintenance of tight junctions, upregulation of anti-apoptotic mediators and downregulation of pro-inflammatory mediators [131,150].

*Flammulina velutipes* polysaccharides significantly improved the signs of UC by the modulation of the expression of some cytokines, genes and proteins of intestinal tight junctions, short-chain fatty acids and intestinal flora control. The physiological responses, mucosal destruction, intestinal ulcers, serum cytokines, and modulation of the gut microbiota were some of the initial pathogeneses from DSS-induced colitis. Treatment with these fungal polysaccharides thus preserved maintenance of the levels of goblet cells, pro-inflammatory cytokines, and nitric oxide and reduced the pathological effects. Tight junction protein synthesis and short-chain fatty acid extents are both mounted in colitis tissue. Additionally, functional prediction of gut microbiota using Phylogenetic Investigation of Communities by Reconstruction of Unobserved States *(PICRUSt*) demonstrated that *Flammulina velutipes* polysaccharide modulated microbial metabolic pathways, which may contribute to the alleviation of ulcerative colitis [112]. Isolated sulphated polysaccharides from *Gracilaria lemaneiformis* introduced mucosal layer healing through reduction of the pro-inflammatory cytokines contributing to colon dysbiosis. It also reduces biochemical marker myeloperoxidase and eventually leads to lowered neutrophils. It maintains the tight junction, thus preventing pathogens to enter [151].

**Figure 6:**
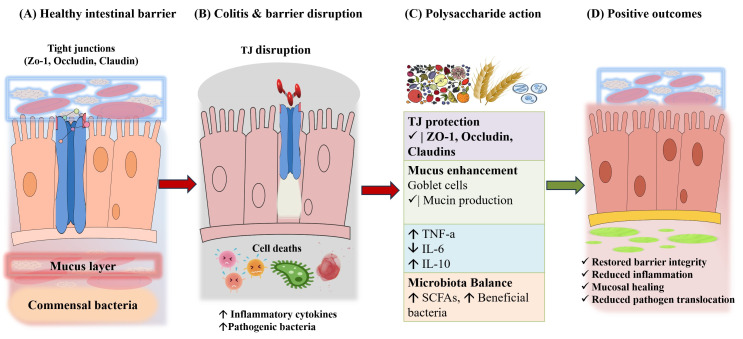
Polysaccharide-mediated intestinal barrier protection. (**A**) Tight junction protection via upregulation of ZO-1, occludin, and claudins. (**B**) Mucus layer enhancement through goblet cell stimulation and increased mucin production. (**C**) Immune modulation characterized by ↓ TNF-α, ↓ IL-6, and ↑ IL-10 and (**D**) Microbiota balance with ↑ SCFAs and ↑ beneficial commensal bacteria.

In mice, UC was caused by 2,4,6-Trinitrobenzenesulfonic acid (TNBS) and treated with extracted purified polysaccharide fractions called HG-2, HG-3, and HG-4 with distinct molecular weights of 12.4, 8.7, and 5.2 kDa, respectively; these were from a traditional medicinal plant known as *Hedysarum* which improved the mice’s body weight and fecal characteristics to varying degrees (*p* < 0.01). In the meantime, to promote intestinal mucosa healing and raise the Colon Macroscopic Damage Index (CMDI) score of UC mice (*p* < 0.05), the macroscopic morphology of the colon and the damage to the colonic mucosa can also be improved to varying degrees. There was a significant increase in HG-4 and a significant drop in tumor necrosis factor-alpha (TNF-α), interleukin-6, and interleukin-10 (*p* < 0.01) [4,44]. In both the DSS-induced rat model and the TNBS-induced mouse model, APS showed significant therapeutic benefits [113]. Baicalin polysaccharide can reduce a neutrophil-derived enzyme activity called myeloperoxidase (MPO), disease activity index (DAI), colonic histological damage, and weight loss in UC mice caused by DSS. Additionally, along with the enhanced expression of proteins like zonula occludens-1 (ZO-1), which are located on the surface of the gut cells lining, occludin and claudin-5 led to the reconstruction of the intestinal barrier [66]. Noni fruit polysaccharide reduces the harm that DSS salt causes to the colonic mucosal barrier by increasing the expression of mucosal and tight junction proteins (ZO-1 and occludin) [152] as explained in Figure 6. Similarly, by lowering serum levels of endotoxin (EDT), diamine oxidase (DAO), and D-lactate (DLA), crude polysaccharides (QHPS) extracts from a two-herb formula consisting of *Lycium barbarum* and *Astragalus membranaceus* promoted the preservation of the integrity of the intestinal mucosa and healed intestinal tract injuries [153].

#### 6.2.2. TLR-Mediated Immunomodulation (NF-κB, MAPK, NLRP3)

Ulcerative colitis is a chronic inflammatory disease in which there is continuous inflammation and breakdown of the colonic mucosa. Its pathogenesis is closely linked with immune dysregulation, overproduction of pro-inflammatory cytokines, disruption of the epithelial barrier, and altered gut microbiota composition. In UC, the inappropriate response of immune cells causes an imbalance in the diagnostic proportion of pro-inflammatory and anti-inflammatory reactions, leading to prolonged mucosal injury and disease progression [153,154]. Cytokines are at the core of the pathophysiology of UC. These are produced by immune system cells like macrophages, T cells, B cells, NK cells, and dendritic cells as well as non-immune cells like epithelial and endothelial cells. Excessive secretion of pro-inflammatory cytokines, such as TNF-α, IL-6, and IL-1β, promotes intestinal inflammation, whereas anti-inflammatory cytokines, such as IL-10, are involved in mucosal healing and immune homeostatic mechanisms [155]. Consequently, therapeutic interventions that focus on cytokine dysregulation and inflammation-related signaling pathways have received significant attention in the management of UC.

**Figure 7:**
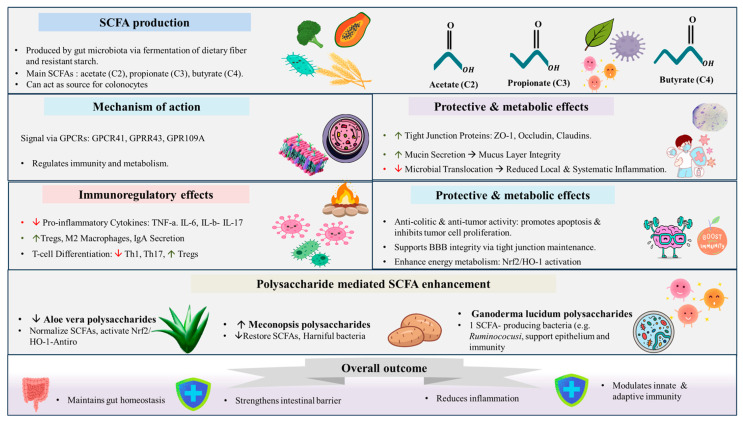
Dietary polysaccharide-mediated short-chain fatty acid (SCFA) production and its immunomodulatory and protective effects on intestinal homeostasis. SCFAs—primarily acetate (C2), propionate (C3), and butyrate (C4)—are produced by the gut microbiota through fermentation of dietary fiber and resistant starch, and butyrate additionally serves as a primary energy source for colonocytes. Mechanistically, SCFAs signal through G-protein-coupled receptors (GPCR41, GPR43, and GPR109A) to regulate immunity and metabolism. Their immunoregulatory effects include suppression of pro-inflammatory cytokines (TNF-α, IL-6, IL-1β, IL-17), promotion of regulatory T cells (Tregs), M2 macrophage polarization, and increased IgA secretion, alongside T-cell differentiation characterized by reduced Th1 and Th17 responses and enhanced Treg populations. Polysaccharide-mediated enhancement of SCFAs has been demonstrated with specific compounds: *Aloe vera* polysaccharides normalize SCFA levels and activate the Nrf2/HO-1 antioxidant pathway, while Meconopsis polysaccharides restore SCFA concentrations and reduce harmful bacteria. These changes lead to upregulation of tight junction proteins (ZO-1, occludin, claudins) and increased mucin secretion, thereby reinforcing mucus layer integrity and reducing microbial translocation, which in turn decreases local and systemic inflammation. Additional protective and metabolic effects include anti-colitic and anti-tumor activity (promoting apoptosis and inhibiting tumor cell proliferation), support of blood–brain barrier (BBB) integrity via tight junction maintenance, and enhanced energy metabolism through Nrf2/HO-1 activation.

At a molecular level, toll-like receptors (TLRs) are very important pattern recognition receptors that are expressed on immune cells such as macrophages, neutrophils, and lymphocytes. Upon binding with polysaccharide ligands, TLRs recruit downstream adaptor molecules such as TNF receptor-associated factor 6 (TRAF6), which in turn activates the regulation of the MAP kinase and NF-κB signaling pathways. These pathways are responsible for the transcription of genes which regulate the development of inflammation and immunity [31]. NF-κB, in particular, is a central transcription factor and modulates the expression of several pro-inflammatory cytokines. Under resting conditions, NF-kappa B is kept in an inactive condition by binding with inhibitory protein, I-kappa B. Upon stimulation, IkB is phosphorylated by IKK, and then NF-κB translocates into the nucleus and induces inflammation and inflammatory genes [106]. Polysaccharides have shown substantial immunomodulatory properties through the regulation of these inflammatory signaling cascades. For example, *Astragalus membranaceus* polysaccharide inhibits an activation of NF-κB, which in turn decreases the synthesis of TNF-α, IL-6, IL-1 beta and other inflammatory mediators. In addition, it modulates the expression of the NFATc protein which reduces the levels of cytokines to a significant extent and mitigates the effects of colitis induced by TNBS in a rat model [156]. Similarly, the polysaccharides obtained from *Eucheuma cottonii* modulate colitis induced by DSS by increasing the level of the anti-inflammatory cytokine IL-10 and decreasing the serum levels of TNF-α, IL-1 and IL-6 [125]. In addition to suppressing cytokines, polysaccharides also affect immune cell differentiation and balance. *Astragalus membranaceus* polysaccharide repairs colonic tissue by modulating the populations of helper T cells and regulatory T cells (Treg). It downregulates Tfh1, Tfh17 and Tfh21 cells and increases the levels of Tfh10 and Treg cells, thereby restoring immune balance and correcting dysbiosis [157] as described in Figure 7. *Tremella fuciformis* polysaccharides also decrease pro-inflammatory cytokines and increase tight junction protein and Foxp3+-T cells, which improve mucosal immunity and intestinal barrier function [114].

MAPK, such as ERK, JNK, and p38 concentration pathways, also perform an essential role in inflammation. Polysaccharides of *Ganoderma lucidum*, *Ganoderma sinense* and *Pleurotus eryngii* have been found to modulate the phosphorylation of the mitogen-activated protein kinases (MAPKs) and nuclear factor B (NF-κB) to affect immune regulation in macrophage and colitis models [106] as mentioned in Table 5.

In turn, the activation of excessive ERK and NF-κB can be reduced by treatment with high doses of polysaccharides, resulting in reduced production of cytokines and attenuation of inflammation in the intestines [35]. Immune imbalance in UC is further characterized by the decrease in Treg cells and the increase in Th17 and Th1 cells in population in gut-associated lymphoid tissue (GALT) with higher production of TNF-α and IL-1 beta. Activation of innate immune pathways, particularly TLR4 signaling, worsens epithelial damage and drives chronic inflammation [110]. Polysaccharides from *Atractylodes macrocephala* and *Poria cocos* have shown therapeutic potential in restoring Th17/Treg homeostasis and inhibiting inflammatory signaling pathways such as IL-6/STAT3 and IL-33/ST2, respectively [128]. Collectively, these findings suggest protective efficiency of polysaccharides in UC through modulation of the infiltration of immune cells, inhibition of pro-inflammatory cytokines, modulation of key immune cell signaling pathways, including NF-κB and MAP kinase, and restoration of immune homeostasis. Through these mechanisms, polysaccharides are responsible for downregulation of mucosal inflammation, enhancement of epithelial integrity and improvement of disease in colitis models.

### 6.3. Oxidative Stress Remodeling via Nrf2/HO-1 Activation

Oxidative stress is an important factor in the pathogenesis of UC, and polysaccharides can counteract this through the antioxidant pathways. In the case of colitis, there are increased markers of oxidative stress in the body which can contribute to intestinal damage while the antioxidant defense systems are impaired [130]. Polysaccharides have protective effects, such as triggering antioxidant signaling pathways and maintaining intestinal barrier integrity. *Aloe vera* belongs to the *Asphodelaceae* family (previously often placed in Xanthorrhoeaceae or Liliaceae). A constant pulpy shrub known as *A. vera* with green leaves, arranged at the stem in a rosette form, is significantly rich in nutrients and various constituents, including glycoproteins, flavonoids, minerals, amino acids, sterols, saponins, and vitamins. It has been widely demonstrated that polysaccharides such as acemannan, pectin and cellulose are prominent active ingredients which are found in *Aloe vera* gel. It has numerous pharmacological activities ranging immune modulating activities, antibacterial, antioxidation, hypolipidemic and anticancer [130].

A previous study has confirmed that through the modification of the janus kinase (JAK)2 signal transducer and activator of transcription (STAT)-3 signaling cascade, *A. vera* significantly improved the colon health of mice. Similarly, aloe polysaccharide showed favorable results, demonstrated by the inhibition of the increased expression of relative genes and interleukins and restoration of the colonic length therefore curing the intestinal colonic injury [160]. *A. vera* polysaccharides also works by invigorating Nrf2/HO-1 signaling cascade. Nrf2 is a nuclear factor erythroid 2-related factor that has a decisive role in the protection of the intestinal layer, activating colonic proteins such as zona occludens and occludin. These factors are sparked off by oxidative stress, which leads to production of inflammatory and antioxidant genes quinine oxidoreductase-1 and heme oxygenase-1. *A. vera* polysaccharides could attenuate the level of these proteins and, on top of that, they can normalize the aberrant level of short-chain fatty acids [142]. *A. vera* polysaccharides activate the Nrf2/HO-1 signaling pathway by reducing the oxidative damage and restoring intestinal barrier proteins such as occludin and zona occludens [142]. *Hericium erinaceus* polysaccharides reduce malondialdehyde and reactive oxygen species, maintain mitochondria membrane integrity, and increase superoxide dismutase activity, thereby promoting the integrity of intestinal cells and reducing oxidative stress [132].

Colitis induced by acetic acid was cured by *Gracilaria caudata* polysaccharide. The effect of 10.0 mg/kg of sulphated polysaccharide treatment was found to be significant. Myeloperoxidase, glutathione peroxidase, and malondialdehyde are three biochemical markers of oxidative stress that, when overexpressed, lead to perturbation of the colon. As a result, the administration of this polysaccharide reduced the increased nitric oxide level in the colon tissue which was confirmed by Western blotting. Additionally, the physical factors such as body weight, colon length and stool consistency were controlled, and *Gracilaria caudata* polysaccharide treated colitis thoroughly, activating a healing process including anti-inflammatory effects [161]. The nanocarrier based on polysaccharides can help the drug to overcome the harsh conditions in the gastrointestinal tract, making it more stable, and also help to concentrate, to the greatest extent possible, in the regions where intestinal inflammation is present. This has the effects of reducing side effects of the drug and increasing the bioavailability of the drug. Specific polysaccharides as prebiotics can give the drug nano-delivery systems the ability to target the colon, depending on the properties of the enzyme. Moreover, they can work together with drugs to decrease the severity of IBD, because of the favorable anti-inflammatory activity and regulation of the intestinal microecology [159]. Some polysaccharide fractions have shown no effect or even pro-inflammatory effects in certain models, and negative results are under-reported in the literature [162].

In summary, the mechanistic categories described in Section 6.1, Section 6.2 and Section 6.3 regarding barrier restoration, microbiota remodeling, SCFA production, immune modulation, and antioxidant effects do not operate in isolation but form an integrated network. As detailed in Section 6.1.2, polysaccharide structure determines which bacterial taxa are enriched, and those taxa determine SCFA profiles [46,117]. SCFAs then act through three parallel arms such as direct epithelial effects (HDAC inhibition → tight junction upregulation), immune effects (GPCR activation → Treg induction, M2 macrophage polarization) and metabolic effects (colonocyte energy substrate) [122,123]. Concurrently, polysaccharides may directly interact with epithelial TLRs, providing microbiota-independent signals that synergize with SCFA-mediated effects. Oxidative stress reduction via Nrf2/HO-1 further amplifies barrier protection [119,140,142]. Thus, the therapeutic efficacy of a given polysaccharide reflects the sum of these interacting pathways, with the relative contribution of each determined by polysaccharide structure, dosage, and host microbiota context.

## 7. Emerging Applications and Innovations

Recent technological and methodological advances have expanded the therapeutic potential of dietary polysaccharides beyond conventional dietary supplementation [2,4,5,163]. This section focuses on four emerging innovation areas that leverage polysaccharide structure and function without reiterating the core mechanisms detailed in Section 5.

Chemical modification has emerged as a powerful approach to enhance therapeutic functions. Among the various modification strategies, sulfation and acetylation have demonstrated promise in colitis models [164,165]. The efficiency of these modifications is critically dependent on structural parameters as the degree and position of sulfation and acetylation have demonstrated particular promise in colitis models [70]. For instance, sulfation of mulberry leaf polysaccharides via sulfation (SMLP) at O-2 and O-6 positions (DS 0.42) preserves the native backbone while introducing negative charges that enhance antimelanoma effects and other biological activities [63]. Similarly, the acetylation at the O-6 position (degree of substitution 0.15) significantly enhances its immunoactivity by activating Dectin-1 and CD14 receptors, demonstrating the capacity of chemical modification to boost biological efficacy [64].

Polysaccharides play a common role in curing colitis through mucus barrier improvement and gut microbiota regulation; when combined with specific beneficial bacteria strains, they may enhance colonization resistance, metabolic output, and therapeutic durability [4]. Polysaccharides such as inulin, fructooligosaccharides, galactooligosaccharides, and pectin are commonly used as prebiotic components in symbiotic formulations. These polysaccharides are selectively fermented by beneficial bacteria strains including *Lactobacillus* and *Bifidobacterium* [4,166]. Black mulberry polysaccharides exhibited a restorative role in enhanced beneficial bacteria growth, along with tight junction protein expression and antioxidant defense integrity. All these improvements impart pronounced anti-colitis functions [133]. Polysaccharides from dietary fiber help the gut beneficial bacteria to produce SCFAs, such as butyrate. These metabolites are energy reservoirs for the colon cells or tissues, serving to prevent inflammation and modulate gut barrier integrity [167].

*Morus atropurpurea Roxb.* polysaccharide accumulation in dextran sodium sulfate (DSS)-induced colitis mice showed recovery with noticeable results, like improved colon damage, improved weight and reduced disease activity index. All these results aided in gut microbiota maintenance and intestinal barrier integrity [165].

Gut microbiota modulation also helps to extinguish inflammatory signaling pathways such as NF-κB and MAPK [133]. In previous study, *Sagittaria sagittifolia L.* polysaccharide PSSP-1 was involved in MAPK/NF-κB signaling pathways, gut beneficial bacteria regulation, inflammation reduction and oxidative stress relief in DSS-induced colitis [126]. Intestinal immunity is distressed by pro-inflammatory cytokines such as TNF-α, IL-1β, IL-6, and IL-8. Polysaccharides inhibit the production of these cytokines by increasing anti-inflammatory cytokines expression, e.g., IL-10 [71]. In a study, rhamnogalacturonan (RGal), belonging to the pectin family, demonstrated a constructive role in impeding inflammation and oxidative stress in a colitis model [168]. Buckwheat polysaccharides are also involved in inflammation obturation and gut beneficial bacteria modulation to control and prevent colitis [134]. Chinese yam polysaccharide has ability to ferment gut microbiota that, in return, improve intestinal anti-inflammatory activity, which was closely associated with microbial fermentation in the gut [107].

SCFAs are also involved in immunity and gene expression regulation via modulation of T-cell differentiation and function [108]. The beneficial bacteria composition and function regulation relays bile acid transformations, stripping the activation of Farnesoid X Receptor (FXR). FXR is an evaluating regulator in lipid metabolism, bile acid synthesis, inflammation expression and glucose homeostasis [31,109].

Natural polysaccharides demonstrate therapeutic potential in severe health disorders including neurological, disorders, lipid metabolism malfunction and obesity other than the GIT-related issues [145]. Nanotechnology is also a green processing technique which can be endorsed to modify polysaccharides with microbes. These eco-friendly microbe-polysaccharide nanomaterials have applications in agriculture, food and pharmaceutical [169]. Polysaccharides have dominant activity over conventional medicines used as effective, therapeutic and safe components [170].

## 8. Comparison with Existing Therapies

Dietary polysaccharides represent a complementary or adjunctive approach for colitis management, rather than a replacement for standard therapies. Current evidence supports their potential as functional food ingredients for long-term supportive care, particularly in maintaining remission and preventing relapse. However, for acute severe flares, standard pharmacotherapies (corticosteroids, biologics) remain the standard of care [165,171]. The existing therapeutic methods to overcome immunity loss and inflammation include biological and small molecule inhibitors, aminosalicylates, corticosteroids and immunoregulators. Immunity recovery and anti-inflammation activity show therapeutic potential in colitis models [172,173]. The conventional strategies and biologics or small-molecular inhibitors are specific in their action against inflammation cascade [174].

Aminosalicylates, e.g., sulfasalazine and mesalamine (5-ASA), are traditionally used for low to moderate ulcerative colitis [172,173]. The therapeutic action of aminosalicylates is to inhibit the inflammation induced by cyclooxygenase (COX) inhibition, lipoxygenase pathways and free radical scavenging in gut lumen [175]. 5-ASAs are suitable for long-term applications due to their high safety profile [176]. For example, an increase in ferroptosis may impede mucosal barrier recovery when subjected to 5-ASA therapy. But the combination of 5-ASA and ferroptosis inhibitors like Fer-1 shows high treatment efficiency [177]. High anti-inflammatory agents like corticosteroids can be used for induced remission in acute flares of UC and CD disorder. They have a broad spectrum of immunosuppressive action [172,173,175]. They also work to bind the glucocorticoid receptor inside immune cells, which in return reacts with master inflammatory transcription factors, e.g., NF-κB and activator protein-1. This helps to inhibit the production of numerous pro-inflammatory molecules in a short time with higher efficiency. Corticosteroids cannot be given to people for long-term use because they can cause diabetes, osteoporosis, HPA-axis suppression and increased susceptibility to infections [173]. Immunomodulatory medication strategies like methotrexate and thiopurines (e.g., azathioprine, 6-mercaptopurine) intend to suppress the immune system more efficiently than corticosteroids. Thus, they can be used for long-term maintenance of remission [172,173,175]. Thiopurines act as “faulty parts” for DNA replication. They avert the clonal expansion of activated T-cells. Patients with corticosteroid-dependent status or who do not qualify for 5-ASA therapy are often subjected to thiopurines [172,173]. They can be used as combination products with biologics to decrease immunogenicity and improve therapy efficiency. But they are slow in action, with some side effects such as hepatotoxicity, myelosuppression, and an increased risk of infections and certain malignancies. This requires continuous treatment monitoring [175]. Medicines like adalimumab, infliximab, and golimumab act on TNF-α (a central pro-inflammatory cytokine) [172,173,175]. TNF-α neutralization stops its binding with its receptors that start inflammatory cascades. These medicines have high treatment efficiency and are known for “silencing” a major arm of the immune assault. They can be given to patients with moderate-to-severe UC and CD for remission induction and maintenance [175]. The side effects of using the drugs can result in the development of anti-drug antibodies (immunogenicity) and primary or secondary loss of response. Polysaccharides have additional therapeutic potential with less side effects as compared to conventional biologics, medicines or small-molecule inhibitors. In addition to health side effects, use of biologics is expensive for long-term applications. For example, biological strategies like anti-TNF therapies (infliximab, adalimumab, golimumab) and IL-23/IL-12 inhibitors (Mirikizumab, Ustekinumab) are highly expensive to treat moderate to severe colitis. These neutralize the specific inflammatory cytokines or Th17/Th1 signaling pathways with a risk of infection [178]. On the other hand, small-molecule therapies like JAK inhibitors and S1P modulators have safety objections with less efficiency as compared to polysaccharides. These small molecules are used as oral drugs, and long-term use can cause lymphopenia [179]. In contrary, polysaccharides potentially boost up the beneficial bacteria production, maintenance and composition, modulate the immune system, and increase barrier integrity, which help attain gut remodeling pathway results [180]. In severe disease conditions, biologics and small molecules are vital for prevention and dietary polysaccharides but risk causing low-toxicity, microbiota-resonant interventions. So, for severe cases, both in combination can help to support mucosal healing, decrease inflammation, and improve long-term disease management [181].

Dietary polysaccharides represent a complementary approach for colitis management, particularly in UC. Preclinical evidence demonstrates consistent modulation of gut microbiota, immune function, and mucosal barrier integrity. However, rigorous clinical trials are needed to establish efficacy, optimal dosing, and long-term safety profiles before these interventions can be recommended as stand-alone therapies. Polysaccharides restore immunity, microbiota functions, and mucosal barrier integrity. The results are supported by various preclinical studies [66,93,112,118,126,131,133,135,136,182] revealing consistent modulation of the TNF-α/pNF-κB/ICAM-1 axis [133], suppression of MAPK/NF-κB signaling [126], inhibition of Th17/Treg imbalance [129], and reinforcement of tight junction proteins, e.g., occludin, claudin-1, and ZO-1 via upregulation of intestinal epithelial transcription factors, e.g., HIF-1α and KLF4 [4]. Polysaccharides like guar gum, pectin, and sulfated mulberry leaf polysaccharide (SMLP), selectively enriched with *Bifidobacterium*, *Lactobacillus*, and *Akkermansia muciniphila*, can inhibit *Enterobacteriaceae* and *Escherichia-Shigella* [165,171]. This combined dual action maintains the gut natural microbiota and decreases the pathogenic microbiota. In short, dietary polysaccharides not only act as colitis therapy but also act as microbiota-resonant, systems-level interventions which may complement pharmacological treatments, demonstrating long-term intestinal immunity and enhanced mucosal healing during colitis. While preclinical evidence shows a promising adjunctive approach, dietary polysaccharides should currently be viewed as potential adjunctive or preventive interventions rather than replacements for established pharmacotherapies. Direct comparative efficacy studies against standard treatments (5-ASAs, biologics) in human trials are lacking.

## 9. Challenges and Knowledge Gaps

Preclinical studies suggest that dietary polysaccharides may offer advantages in terms of safety and multi-targeted mechanisms; direct comparative efficacy studies against conventional therapies (e.g., 5-ASAs, anti-TNF biologics) are lacking. Currently, there is insufficient evidence to claim superior efficacy; rather, polysaccharides should be viewed as potential adjunctive or complementary interventions requiring rigorous head-to-head clinical trials. Still there are some challenges which can be faced if the application is without mechanistic and clinical monitoring. Natural polysaccharides are complex and diverse. Due to the diverse nature, the knowledge of targeted bioactive compounds is necessary in order to overcome side effects. Nevertheless, not all polysaccharides exert uniform beneficial effects. High-molecular-weight, insoluble polysaccharides may exacerbate symptoms during active disease, whereas low-molecular-weight, soluble fractions demonstrate greater efficacy. Moreover, these effects are highly context-dependent, varying according to polysaccharide structure, dosage, host microbiota composition, and disease stage. Moreover, metabolite production after beneficial bacteria fermentation in the gut and its targeting side effect can affect cumulative disease control results. The pre-existing natural microbiota in individual bodies can be affected by polysaccharides that are introduced without optimization. The animal model limitations for clinical research hinder the future work for microbiome diversity and human disease pathophysiology, jeopardizing translational relevance. The ”Remodeling” concept is not under strict observation, lacking standard definition and giving debatable endpoints from mucosal healing to immune reprogramming. In the end, experiment optimization for pharmaceutical and therapeutic use is needed, along with a daunting regulatory pathway from a dietary component for certified and approved therapy. All these limitations demand humanized practical models, gnotobiotic, polysaccharide libraries, and multi-omics technologies optimization. There is also a need to standardize microbiome-stratified trials before supplement or drug approval for IBD.

## 10. Limitations

Despite the preclinical evidence presented, several critical limitations warrant acknowledgment. Most studies investigating dietary polysaccharides in colitis have been conducted in rodent models, primarily using DSS- or TNBS-induced colitis. Although informative, these models do not fully recapitulate the heterogeneous, relapsing-remitting nature of human ulcerative colitis. Acute chemically induced models lack the chronicity, genetic susceptibility, and environmental triggers characteristic of human inflammatory bowel disease. Furthermore, direct comparative efficacy data between polysaccharides and standard pharmacotherapies—such as 5-aminosalicylates (5-ASAs), anti-tumor necrosis factor biologics, and Janus kinase inhibitors—from human trials are absent. Consequently, claims of superior or equivalent efficacy remain speculative. The structural heterogeneity of natural polysaccharide extracts poses substantial challenges for reproducibility and clinical translation. Batch-to-batch variation in molecular weight, degree of branching, and monosaccharide composition complicates the establishment of consistent structure–activity relationships across studies. Additionally, while the gastrointestinal remodeling concept is conceptually useful, it lacks standardized histopathological and molecular endpoints, thereby hindering cross-study comparisons of remodeling outcomes.

Addressing these limitations will require well-designed, randomized controlled human trials using standardized polysaccharide preparations, integrated multi-omics profiling, and long-term follow-up. To date, no randomized controlled trials evaluating any specific polysaccharide for UC have been published. The available evidence remains entirely preclinical, confined to murine models of DSS-induced colitis, in vitro cell culture systems (primarily Caco-2 monolayers), and in vitro fermentation studies using human fecal inoculum. This absence of clinical evidence underscores the urgent need for well-powered, placebo-controlled randomized controlled trials to validate the safety and efficacy of these promising natural compounds in human UC patients before they can be considered viable therapeutic alternatives to conventional treatments.

Regardless of the preclinical evidence presented, several critical limitations must be acknowledged. Many studies on dietary polysaccharides in colitis have been conducted in rodent models (primarily DSS- or TNBS-induced colitis) which, while informative, do not fully recapitulate the heterogeneous, relapsing-remitting nature of human ulcerative colitis. Acute chemical-induced models lack the chronicity, genetic susceptibility, and environmental trigger attributes of human IBD. The direct comparative efficacy data between polysaccharides and standard pharmacotherapies (e.g., 5-ASAs, anti-TNF biologics, JAK inhibitors) in human trials are absent; thus, claims of superior or equivalent efficacy remain speculative. The structural heterogeneity of natural polysaccharide extracts poses significant challenges for reproducibility and clinical translation. Batch-to-batch variation in molecular weight, degree of branching, and monosaccharide composition complicates the establishment of consistent structure–activity relationships across studies. The gastrointestinal remodeling concept is conceptually useful but lacks standardized histopathological and molecular endpoints, making cross-study comparisons of remodeling outcomes difficult. Addressing these limitations, future work will require well-designed, randomized controlled human trials with standardized polysaccharide preparations and multi-omics profiling along with long-term follow-up.

## 11. Future Directions

Recently, GIT remodeling by dietary polysaccharides has been investigated primarily through observational and correlative studies. To achieve enhanced efficacy in alleviating colitis, future research should adopt mechanism-driven and optimized frameworks to standardize the management and use of dietary polysaccharides. A paradigm shift is required, moving away from crude extract applications toward the use of chemically well-defined polysaccharide structures. This effort should emphasize advanced glycoanalytical, synthetic biology approaches, and chemoenzymatic synthesis to generate precise oligosaccharide and polysaccharide structures. Such standardization will facilitate the elucidation of structure–function relationships, specifically regarding degrees of polymerization, identification of specific glycosidic linkages, and substitution patterns that optimally induce host gut and microbial functions. Second, mechanistic insights should guide the design of “smart” intervention strategies. This can be accomplished through engineered polysaccharide blends or synbiotic combinations. Intervention strategies along with standardization of dose must clarify the specific mechanism by which each microbial strain acts upon specific polysaccharides to produce distinct metabolites. Third, microbiome-directed nutrition, integrated with optimized polysaccharide dosing, should be combined with multi-omics strategies. Diagnostic and predictive biomarkers can be derived from patients’ multi-omics data. Such biomarkers may serve as constructive tools to characterize the gut microbial composition of individual patients prior to polysaccharide dose induction.

Future studies should systematically address batch-to-batch variation from natural sources, standardization of extraction protocols, validate purity (protein/phenolic removal), and ensure reproducibility across studies, all of which warrant dedicated investigation. Concurrently, lack of sufficient human or animal studies, gut induction models, and patient-derived microbiota strain libraries are needed to enhance the efficacy of polysaccharides relative to conventional drugs. Clinical trials must support the targeted dietary polysaccharide beyond the generalized concept that “dietary fiber is good” to targeted, clinically validated, research-driven polysaccharide therapies that hold promise for benefiting gut-related health issues and providing long-term remission from colitis.

## 12. Conclusions

Dietary polysaccharides remodel the inflammation of the gastrointestinal tract in colitis through three interconnected mechanisms: enrichment of beneficial microbiota (*Lactobacillus*, *Bifidobacterium*, *Akkermansia*) while suppressing pathogenic *Enterobacteriaceae*, production of barrier-strengthening SCFAs (particularly butyrate), and suppression of NF-κB/NLRP3 inflammatory signaling. These effects are structure-dependent, with optimal activity at low molecular weight and branching degrees above 0.3. Fermentation produces SCFAs, particularly butyrate, which reinforce epithelial barrier integrity by upregulating tight junction proteins, e.g., ZO-1, occludin, and claudin, through histone deacetylase inhibition. Polysaccharides exert immunomodulatory control by suppressing NF-κB, MAPK, and NLRP3 inflammasome signaling, with sulfation (DS 0.2–0.5) and acetylation (DS 0.1–0.3) conferring enhanced or pathway-specific anti-inflammatory activity. However, oxidative stress is counteracted through Nrf2/HO-1 pathway activation, reducing reactive oxygen species and protecting colonocytes from injury. These structure-dependent mechanisms show superior bioavailability and faster action: β-1,3/β-1,6 glucans excel at immunomodulation, and pectin-type polysaccharides optimize barrier protection. However, current evidence is entirely preclinical, derived from murine models and in vitro systems, with no human trials validating efficacy or safety. While preclinical evidence consistently demonstrates these multi-target effects, direct structure–mechanism correlations need validation in human trials. Collectively, dietary polysaccharides function not as passive fibers but as bioactive, structure-guided modulators capable of restoring intestinal homeostasis in colitis.

## Figures and Tables

**Table 1:** Key biomarkers for assessing GI remodeling.

Biomarker	Biological/Clinical Relevance	Assessment Type	Evidence & Utility	References
Fecal calprotectin	Reflects neutrophil infiltration and mucosal inflammation	Non-invasive stool marker	High sensitivity for detecting active colitis; AUC ranges 0.70–0.99 in UC and CD for mucosal inflammation	[25]
Fecal MMP-9	Indicator of ECM proteolysis	Fecal ELISA	Correlates with endoscopic ulceration; sensitivity ~90% in CD and UC; reflects active ECM degradation	[28]
Collagen degradation biomarkers (C3M, C4M)	Products of MMP-mediated ECM breakdown	Serum neo-epitope assays	Elevated in active CD & UC; combination markers show high AUC for activity discrimination	[26]
Collagen formation biomarkers (PRO-C3, PRO-C4, PRO-C5)	Reflect collagen synthesis and ECM remodeling	Serum neo-epitope assays	Indicative of ECM turnover/healing; formation markers correlate with disease severity and fibrosis	[26]
CRP (C-reactive Protein)	General systemic inflammatory marker	Serum	Commonly elevated in active disease; correlates with inflammation and progression	[25]
Serum ECM fragments	Surrogates of transmural remodeling/fibrosis	Blood biomarkers	Various ECM fragments (e.g., collagen fragments) correlate with disease burden and remodeling	[29]
Lactoferrin	Neutrophil-related intestinal inflammation marker	Stool	Elevated levels in active colitis; used with calprotectin for inflammation detection	[28]
Lipocalin-2 (NGAL)	Associated with neutrophil activation and epithelial injury	Stool/serum	Correlates with disease activity and severity; potential adjunct marker	[28]
Matrix metalloproteinases (MMPs)	Direct ECM degrading enzymes	Tissue/serum/stool	MMP-mediated ECM breakdown influences remodeling; increased proteolytic activity observed in colitis	[17]

Abbreviations: GI, Gastrointestinal; ECM, Extracellular Matrix; MMP-9, Matrix Metalloproteinase-9; MMPs, Matrix Metalloproteinases; ELISA, Enzyme-Linked Immunosorbent Assay; C3M, Matrix Metallinase-mediated Type III Collagen Degradation Fragment; C4M, Matrix Metalloproteinase-mediated Type IV Collagen Degradation Fragment; PRO-C3, N-terminal Propeptide of Type III Collagen; PRO-C4, N-terminal Propeptide of Type IV Collagen; PRO-C5, N-terminal Propeptide of Type V Collagen; CRP, C-Reactive Protein; NGAL, Neutrophil Gelatinase-Associated Lipocalin; CD, Crohn’s Disease; UC, Ulcerative Colitis; AUC, Area Under the Curve.

**Table 2:** Comprehensive structure–function relationships of dietary polysaccharides in colitis.

Polysaccharide (Source)	Structural Features	Molecular Mechanisms	Biological Effects	Key References
*Astragalus membranaceus* polysaccharide (APS-I)	MW: >2000 kDa Composition: Man, Rha, GlcA, GalA, Glc, Gal, Xyl, Ara Linkages: Complex heteropolysaccharide Branching: High Modifications: Native	Stimulates macrophage proliferation ↑Cytokine secretion TLR4-mediated activation	Immunostimulation Antioxidant activity Cardio-protective effects	[56,57]
*Astragalus membranaceus* polysaccharide (APS-II)	MW: ~10 kDa Composition: Similar to APS-I but different molar ratios Linkages: Conserved backbone Branching: Moderate Modifications: Native	Differential immune cell activation Moderate cytokine induction Balanced Th1/Th2 response	Moderate immunomodulation Potential for sustained therapy	[56]
*Astragalus membranaceus* polysaccharide (APS-III)	MW: ~300 Da (oligosaccharide) Composition: Short-chain fragments Linkages: Degraded linkages Branching: Minimal (linear) Modifications: Acid/enzymatic hydrolysis	Rapid absorption Direct epithelial interaction Low immunogenicity	Fast-acting High bioavailability Potential for acute intervention	[56]
*Astragalus membranaceus* APS2-I	MW: 1.96 × 10^6^ Da (1960 kDa) Composition: Man, Rha, GlcA, GalA, Glc, Gal, Xyl, Ara (specific molar ratios) Linkages: Complex heterogeneous Branching: Very high Modifications: Native	Strong antioxidant activity ↑Superoxide dismutase ↓Malondialdehyde Cardio-protective signaling	Significant antioxidant effects Cardio-protective activity Reduces oxidative stress markers	[57]
*Astragalus membranaceus* APS3-I	MW: 3.91 × 10^6^ Da (3910 kDa) Composition: Similar monosaccharide profile Linkages: Complex, potentially different backbone Branching: Very high Modifications: Native	Potent free radical scavenging ↑Glutathione peroxidase ↓Reactive oxygen species	Superior antioxidant capacity Strong cardio-protection Highest MW fraction shows maximal activity	[57]
*Astragalus membranaceus* low-MW APS (LMW-APS)	MW: 2.60–8.38 kDa (degraded fragments) Composition: Retained core monosaccharides Linkages: Preserved structural integrity Branching: Reduced from native Modifications: Acid/enzymatic hydrolysis	Restores SCFA production Regulates Th17/Treg homeostasis Microbiota-dependent mechanism ↑ZO-1, occludin, claudin	Alleviates DSS-induced colitis Enhanced bioavailability vs. native Restores intestinal barrier Modulates gut microbiota	[12,65]
*Astragalus membranaceus* arabinogalacta*n* (Type I)	MW: Variable Composition: β-1,4-Galp backbone with arabinose branches Linkages: β-1,4 galactan core Branching: α-1,5-Araf side chains Modifications: Native	Immunomodulation via specific backbone recognition ↑Macrophage activation ↓Pro-inflammatory cytokines	Critical for immunomodulatory activity Backbone configuration essential for function	[60]
*Astragalus membranaceus* arabinogalactan (Type II)	MW: Variable Composition: β-1,3/β-1,6-Galp backbone with arabinogalactan side chains Linkages: β-1,3 and β-1,6 galactan Branching: Very high (DB > 0.4) Modifications: Native	Enhanced immune recognition Dectin-1 binding ↑TNF-α, IL-6, IL-10	Superior immunomodulatory activity vs. Type I Side chain distribution critical for function	[60]
*Scutellaria baicalensis* polysaccharide	MW: 18.3, 42.7, and 156.2 kDa Composition: Rha, GalA, Glc, Gal, Ara Linkages: 1,4-α-D-GalpA backbone with 1,2,4-Rhap inserts Branching: Highly branched rhamnogalacturonan (RG-I) structure Modifications: Native	Suppresses NF-κB signaling ↓NLRP3 inflammasome activation ↓Caspase-1, ↓IL-1β ↑ZO-1, occludin, claudin-5	Anti-inflammatory Reduces MPO activity and DAI Reconstructs intestinal barrier Effective in DSS-induced UC	[58,66]
Chrysanthemum morifolium polysaccharide	MW: 23.8, 56.4, 128.7 kDa Composition: Ara, Gal, Glc, Rha, GalA Linkages: 1,5-Ara, 1,3,5-Ara, 1,4-Gal, terminal Gal Branching: Pectic arabinogalactan with complex branching (DB ~0.35) Modifications: Native	Blocks NF-κB and JAK/STAT pathways ↓TLR4 expression ↓IL-6/JAK2/STAT3 signaling ↑ *Butyricicoccus*, Clostridium, *Lactobacillus*	↓IL-23, TNF-α, IFN-γ Preserves gut microbial balance ↓*Escherichia*, *Enterococcus* Ameliorates colitis in rats	[58]
*Lycium barbarum* polysaccharide	MW: 10–200 kDa Composition: Gal, Rha, Ara, Man, Glc, GalA (high uronic acid content) Linkages: Complex pectic polysaccharide with RG-I domains Branching: High Modifications: Native	Sustained fermentation profile ↑SCFA production (especially butyrate) ↑ *Lactobacillus*, *Bifidobacterium* Antioxidant via Nrf2/HO-1	Strong prebiotic functionality Sustained release in colon Neuroprotective and immunomodulatory effects	[58]
*Polygonatum sibiricum* PSO-A (acetylated fructooligosaccharide)	MW: 2.12 kDa (lowest among active polysaccharides) Composition: Fru, Glc (Fru:Glc = 8:1) Linkages: Linear β-2,1 fructan (inulin-type) Branching: Linear (no branches) Modifications: Acetylated at O-6 position (DS 0.15)	Activates Dectin-1 and CD14 receptors Direct NLRP3 inflammasome inhibition ↓ASC oligomerization ↓caspase-1 activation Does not affect NF-κB pathway	Unique NLRP3-specific mechanism Alleviates DSS-induced colitis Acetylation essential for enhanced immunoactivity Superior to non-acetylated version	[64,67]
*Polygonatum kingianum* polysaccharide	MW: 15.8, 42.3, 98.6 kDa (multiple fractions) Composition: Fru, Glc (Fru: Glc = 6:1 to 12:1) Linkages: β-2,6 fructan backbone with β-2,1 branch. Branching: Moderate (branched fructan) Modifications: Native	Modulates gut microbiota composition ↑SCFA-producing genera (*Roseburia*, *Faecalibacterium*) Restores tryptophan metabolism ↓Pro-inflammatory cytokines	Anti-diabetic effects Immunomodulatory activity Ameliorates metabolic disorders Regulates oxidative stress networks	[67,68]
Ginseng polysaccharide	MW: 10–150 kDa (heterogeneous, broad distribution) Composition: Glc, Gal, Ara, Rha, GalA Linkages: Starch-like (α-1,4, α-1,6) + pectin domains Branching: Variable (dual-domain structure) Modifications: Native	Diverse host physiological interactions ↑Lactobacillus, Bifidobacterium Regulates tryptophan metabolism ↓Pro-inflammatory cytokines	Broad-spectrum biological activities Ameliorates UC via gut microbiota Improves barrier function Anti-fatigue and neuroprotective effects	[69]
Black mulberry polysaccharide	MW: 31.5–156.8 kDa (broad distribution) Composition: Glc, Gal, Ara, Rha, GalA, Man, Xyl Linkages: Complex pectic polysaccharide with RG-I domains Branching: High (DB 0.38) Modifications: Native	TNF-α/pNF-κB/ICAM-1 pathway inhibition ↑Antioxidant defense (SOD, CAT, GSH-Px) ↑Tight junction proteins (ZO-1, occludin) ↑ beneficial bacteria growth	Potent antioxidant activity Anti-colitis function Enhanced beneficial bacteria (*Lactobacillus*, *Bifidobacterium*) Superior to non-mulberry polysaccharides	[59]
Sulfated mulberry leaf polysaccharide	MW: 42.3 kDa (after sulfation; native ~same range) Composition: Glc, Gal, Ara, Rha, Xyl, Man Linkages: Preserved native pectic backbone Branching: High (DB preserved) Modifications: Sulfation at O-2 and O-6 positions (DS 0.42)	Enhanced TLR4/MD2 binding (vs. native) ↓Myd88 recruitment Superior NF-κB suppression Selective microbial enrichment	Enhanced anti-melanoma effects vs. native Enhanced anti-colitis activity Selective enrichment of *Akkermansia*, *Lactobacillus* ↓*Enterobacteriaceae* Negative charges critical for activity	[63,70]
Kiwifruit polysaccharide	MW: 68.4, 125.7 kDa Composition: Glc, Gal, Ara, Rha, GalA, Xyl Linkages: Pectin-type with RG-I domains Branching: High (DB 0.32) Modifications: Native	Gut microbiota-dependent fucosylation induction ↑IL-22 production ↑ZO-1, occludin, claudin-3 Regulates tryptophan metabolism	Reduces DSS-induced UC symptoms ↑SCFA concentrations in cecum Promotes colon fucosylation Improves metabolic health	[58]
Alfalfa polysaccharide	MW: 22.6, 48.3, 95.7 kDa Composition: Glc, Gal, Ara, Rha, Xyl, Man Linkages: Arabinogalactan type II Branching: Moderate (DB 0.22) Modifications: Native	TLR4/MyD88/NF-κB pathway inhibition ↑ *Lachnospiraceae*, Parabacteroides distasonis ↑Goblet cell count ↓MUC2, MUC5AC (normalized)	Improves DSS-induced intestinal damage Protects intestinal integrity ↑ZO-1, occludin, claudin-1 Restores normal mucin expression	[58]
Sagittaria *sagittifolia* polysaccharide (PSSP-1)	MW: 28.4 kDa Composition: Glc, Gal, Ara, Rha, Man, Xyl, GalA Linkages: 1,4-α-D-GalpA backbone with 1,2,4-Rhap inserts Branching: High (rhamnogalacturonan type) Modifications: Native	MAPK/NF-κB signaling suppression (↓p-ERK, ↓p-JNK, ↓p-p38) ↓COX-2, iNOS ↑Gut beneficial bacteria	Mitigates DSS-induced colitis Relieves oxidative stress Modulates gut microbiota Prevalence of RG structures in medicinal plants	[58]
*Hedysarum* polysaccharide (HG-2)	MW: 12.4 kDa Composition: Glc, Gal, Ara Linkages: α-1,4 Glc, α-1,6 Gal Branching: Highest among HG fractions (DB ~0.30) Modifications: Native	↓TNF-α, IL-6 ↑IL-10 (moderate) ↓CMDI scores	Improves body weight and fecal characteristics Moderate mucosal healing Less effective than HG-4	[56]
*Hedysarum* polysaccharide (HG-3)	MW: 8.7 kDa Composition: Glc, Gal, Ara Linkages: α-1,4 Glc, α-1,6 Gal Branching: Moderate (DB ~0.22) Modifications: Native	↓TNF-α, IL-6 ↑IL-10 (better than HG-2) ↓CMDI scores	Intermediate efficacy Better than HG-2, less than HG-4 Structure compactness influences accessibility	[56]
*Hedysarum* polysaccharide (HG-4)	MW: 5.2 kDa (lowest) Composition: Glc, Gal, Ara Linkages: α-1,4 Glc, α-1,6 Gal Branching: Lowest (DB ~0.15)—most linear Modifications: Native	↓TNF-α, IL-6 (most effective) ↑IL-10 (highest) ↓CMDI scores (best improvement)	Best mucosal healing among HG fractions Superior to higher MW counterparts Lowest MW shows highest activity	[56]
Noni fruit polysaccharide	MW: 35.2 kDa Composition: Glc, Gal, Ara, Rha, Man, GalA Linkages: Type II arabinogalactan Branching: High (DB 0.41) Modifications: Native	↑ZO-1 and occludin expression ↓Endotoxin, diamine oxidase, D-lactate Protects mucosal barrier Immunostimulatory effects	Reduces DSS-induced colonic mucosal barrier damage Improves intestinal permeability High branching contributes to immunostimulant	[58]
Chinese yam polysaccharide	MW: 46.7 kDa Composition: Glc, Gal, Man, Ara, Rha Linkages: α-1,4 Glc (starch-like) + pectin domains Branching: Moderate (dual-domain architecture) Modifications: Native	Gut microbial fermentation-dependent activity ↓Pro-inflammatory cytokines ↑SCFA production (butyrate, propionate) Fermentation products mediate effects	Intestinal anti-inflammatory activity requires intact microbiota No effect in germ-free mice Dual role: energy source + bioactive modifier	[58]
Buckwheat polysaccharide—FTP	MW: 68.3 kDa Composition: Glc, Gal, Ara, Man, Xyl (Glc-rich) Linkages: β-1,3/β-1,6 glucan (fungal-type structure from plant) Branching: High (glucan branching) Modifications: Native	Inflammation obturation Gut beneficial bacteria modulation ↓NF-κB, MAPK ↑ *Lachnospiraceae*, *Ruminococcaceae*	Controls and prevents colitis Less effective for barrier protection vs. FEP Coexisting polysaccharides offer multi-target approach	[58]
Buckwheat polysaccharide—FEP	MW: 42.7 kDa Composition Glc, Gal, Ara, Rha (pectin-type) Linkages: Pectic with RG domains Branching: Moderate Modifications: Native	Barrier protection (superior to FTP) ↑Tight junction proteins ↑Mucus secretion ↓Pro-inflammatory cytokines	More effective than FTP for barrier protection Synergistic effects with FTP Optimal to use mixed fractions	[58]
Water-soluble polysaccharides	MW: Variable Composition: Galactose and rhamnose-rich variants show high uronic acid Linkages: Specific glycosidic linkages determine bioactivity Branching: Pectic domains as key recognition sites Modifications: Native	Pectic domains serve as immune receptor recognition sites High uronic acid = sustained fermentation Specific linkages dictate prebiotic selectivity	Strong prebiotic functionality Immune receptor recognition Sustained fermentation profiles Source-dependent bioactivity	[58,61]

Here in the table upword arrow (↑) indicates significantly upregulated/increased expression or enhanced biological effect; while downword arrow (↓) indicates significantly downregulated/decreased expression or attenuated biological effect.

**Table 4:** Role of short-chain fatty acids (SCFAs) in gut health, immune regulation, and colitis.

Category	Active Compounds	Key Mechanisms	Biological and Clinical Effects	References
Receptor-mediated signaling	SCFAs	Activation of GPR41, GPR43, GPR109A; inhibition of histone deacetylases (HDACs)	Regulation of inflammatory responses, metabolism, and immune functions	[120,141]
Anticancer and cytoprotective roles	SCFAs; butyrate-producing bacteria	Promotion of apoptosis; inhibition of tumor cell proliferation; receptor-mediated signaling	Reduced risk of colitis-associated colon cancer	[122,124]
Polysaccharide-mediated SCFA enhancement	*Aloe vera*, *Meconopsis*, *Ganoderma lucidum*, Almond polysaccharides	Restoration of SCFA levels; modulation of gut microbiota; activation of Nrf2/HO-1 signaling	Improved intestinal barrier function, antioxidant defense, and immune regulation in UC models	[119,140,142]
Types and production	Acetate (C2), Propionate (C3), Butyrate (C4); microbial fermentation of dietary fiber and resistant starch	Serve as signaling molecules and primary energy source for colonocytes	Maintenance of colonic energy metabolism and gut homeostasis	[135,136]
Immunomodulatory effects	SCFAs, particularly butyrate	Modulation of dendritic cells, T cells (Th1, Th17, Tregs), and B cells via HDAC inhibition and GPCR signaling	Enhanced immune tolerance, increased IgA production, controlled inflammation	[122,123]
Intestinal barrier integrity	SCFAs	Induction of IL-18, mucin and antimicrobial peptide secretion; upregulation of tight-junction proteins	Strengthening of epithelial barrier and reduction of microbial translocation	[143]
Gut–brain barrier regulation	SCFAs	Regulation of tight-junction protein expression	Preservation of blood–brain barrier integrity and reduction of neuroinflammation	[144]
Anti-inflammatory actions in colitis	Butyrate	Suppression of pro-inflammatory cytokines (IL-6, IL-17, TNF-α, IL-1β); inhibition of Th17 cells via Sirtuin-1/mTOR; promotion of M2 macrophage polarization	Attenuation of experimental colitis and intestinal inflammation	[92,145]

**Table 5:** Immunomodulatory effects and mechanisms of polysaccharides in ulcerative colitis.

Category	Active Polysaccharide/Component	Molecular Mechanisms	Biological and Clinical Effects	References
Pathophysiology & cytokines	TNF-α, IL-6, IL-1β (Pro-inflammatory); IL-10 (Anti-inflammatory)	Imbalance of immune cell activation (Macrophages, T cells, B cells, NK cells, Dendritic cells)	Chronic inflammation, mucosal damage, and disease progression in UC	[153,158]
TLR & NF-κB signaling	Polysaccharide ligands	Activation of TLRs and TRAF6; trigger of MAPK and NF-κB pathways; translocation of NF-κB to nucleus	Regulation of transcription for genes involved in inflammation and immunity	[31,106]
Cytokine modulation	*Astragalus membranaceus* polysaccharide	Suppression of NF-κB activation; modulation of NFATc protein expression	Reduction of TNF-α, IL-6, and IL-1β; improvement of TNBS-induced colitis	[156]
Anti-inflammatory induction	*Eucheuma cottonii* polysaccharides	Suppression of pro-inflammatory serum levels; enhancement of anti-inflammatory pathways	Increase in IL-10; attenuation of DSS-induced colitis	[125]
T-cell balance & differentiation	*Astragalus membranaceus* polysaccharide	Downregulation of Tfh1, Tfh17, and Tfh21; upregulation of Tfh10 and Treg cells	Restoration of immune balance; repair of colonic tissue; correction of dysbiosis	[157]
Barrier & mucosal immunity	*Tremella fuciformis* polysaccharides	Upregulation of tight junction proteins and Foxp3^+^ T cells	Reduction of pro-inflammatory cytokines; enhanced intestinal barrier function	[131,159]
MAPK & macrophage regulation	*Ganoderma lucidum*, *Ganoderma sinense*, *Pleurotus eryngii*	Modulation of MAPK (ERK, JNK, p38) and NF-κB phosphorylation	Enhanced immune regulation in macrophage and colitis models	[35,106,127]
Th17/Treg homeostasis	*Atractylodes macrocephala*, *Poria cocos*	Inhibition of IL-6/STAT3 and IL-33/ST2 signaling pathways	Restoration of Th17/Treg balance; inhibition of innate immune damage (TLR4)	[128,157]

## Data Availability

No new data was created or analyzed in this study. Data sharing is not applicable to this article.

## Abbreviations

The following abbreviations are used in this manuscript:

5-ASA	5-Aminosalicylic acid
CD	Crohn’s disease
DAI	Disease activity index
DB	Degree of branching
DS	Degree of Substitution
DSS	Dextran sulfate sodium
ECM	Extracellular matrix
GIT	Gastrointestinal Tract
HDAC	Histone deacetylase
HO-1	Heme oxygenase-1
IBD	Inflammatory bowel disease
IECs	Intestinal epithelial cells
JAK	Janus kinase
MAPK	Mitogen-activated protein kinase
MMPs	Matrix metalloproteinases
MPO	Myeloperoxidase
MW	Molecular weight
NF-κB	Nuclear factor-kappa B
NLRP3	Nucleotide-binding domain leucine-rich repeat pyrin-containing protein 3
Nrf2	Nuclear factor erythroid 2-related factor 2
SCFAs	Short-chain fatty acids
STAT	Signal transducer and activator of transcription
TJs	Tight junctions
TLRs	Toll-like receptors
TNBS	2,4,6-Trinitrobenzenesulfonic acid
Tregs	Regulatory T cells
UC	Ulcerative colitis
ZO-1	Zonula occludens-1
